# Triterpenoid saponins from the aerial parts of *Phytolacca americana* and their anti-influenza A virus activity

**DOI:** 10.1098/rsos.262034

**Published:** 2026-11-04

**Authors:** Shuo Tian, Jisu Park, You Jin Kim, Sainan Li, Wonkyun Ronny Im, Sung Ho Jeon, MinKyun Na

**Affiliations:** ^1^ College of Pharmacy, Chungnam National University, Daejeon, Republic of Korea; ^2^ Department of Life Science and Multidisciplinary Genome Institute, Hallym University, Chuncheon, Republic of Korea; ^3^ Hallym AI-Biohealth R&BD Center, Hallym University, Chuncheon, Republic of Korea

**Keywords:** *Phytolacca americana*, phytolaccagenin, phytolaccagenic acid, influenza A virus, antiviral activity

## Abstract

*Phytolacca americana* L. (Phytolaccaceae), a perennial species native to North America, is rich in oleanane-type triterpenoid saponins. In this study, ultra-performance liquid chromatography coupled with quadrupole time-of-flight mass spectrometry (UPLC–QTOF–MS) profiling of the aerial-part extract revealed characteristic fragment ions of phytolaccagenin- and phytolaccagenic acid-type saponins with diverse glycosylation. MS-guided annotation enabled targeted isolation and structural elucidation of two previously undescribed compounds, one oleanane-type triterpenoid saponin (**1**) and one neolignan (**3**). Additionally, compound **2**, which had been reported only based on mass spectrometric data, was isolated, and its structure was fully elucidated for the first time, together with those of 10 known oleanane-type triterpenoid saponins (**4**–**13**). Their structures were elucidated by comprehensive one-dimensional and two-dimensional nuclear magnetic resonance spectroscopic analyses combined with UPLC–QTOF–MS data. Although *P. americana* extracts have been reported to inhibit influenza A virus infection in previous studies, the bioactive constituents responsible for this antiviral activity have not been identified. The crude extract and isolated saponins were evaluated using complementary *in vitro* assays. In H1N1-infected A549 cells, compound **11** (3-*O*-trisaccharide phytolaccagenic acid) reduced viral NS1 RNA levels at 6 and 24 hpi as determined by RT–qPCR. Antiviral activity was further confirmed by plaque reduction and immunoblot analyses, with a plaque-assay IC_50_ of 57.98 ± 0.26 µM.

## 1. Introduction

The genus *Phytolacca* (Phytolaccaceae) comprises approximately 22 species distributed across Asia and the Americas. *Phytolacca americana* L., commonly known as American pokeweed, is a perennial herbaceous plant and native to North America. It is widely distributed across temperate regions, including North America, Asia and Europe [1]. Phytochemical studies have shown that *P. americana* is rich in oleanane-type triterpenoid saponins, including esculentosides and phytolaccosides, which are characteristic and predominant metabolites of this genus [2]. Previous phytochemical investigations of *P. americana* have focused primarily on the roots, leading to the isolation and characterization of numerous triterpenoids, particularly triterpenoid saponins [3]. By contrast, phytochemical studies on the aerial parts have been limited.

*Phytolacca americana* has traditionally been used to relieve fever, sore throat, lymphadenitis and inflammatory swelling, symptoms that are partially associated with upper-respiratory viral infections [2,4]. Notably, oleanane-type triterpenoid saponins are widely recognized for broad pharmacological profiles, including anti-inflammatory and immunomodulatory effects, and selected saponin skeletons have been reported to exhibit antiviral activity [5–7]. Moreover, a previous study reported that crude leaf extract of *P. americana* inhibited influenza A virus (IAV) infection [8]; however, this finding was limited to the extract level, and the bioactive constituents responsible for the antiviral effect were not identified. To date, phytochemical investigations into the potential anti-IAV properties of *P. americana* are unavailable, and the antiviral relevance of its triterpenoid saponins has not been elucidated.

This study reports a comprehensive characterization of triterpenoid saponins from the aerial parts of *P. americana*. Phytochemical profiling using ultra-performance liquid chromatography coupled with quadrupole time-of-flight mass spectrometry (UPLC–QTOF–MS) identified multiple fragment ions corresponding to phytolaccagenin- or phytolaccagenic acid-type saponins, displaying diverse glycosylation patterns composed of hexose, deoxyhexose and pentose residues. These MS fragment ion patterns enabled tentative annotation of several previously unreported saponins (e.g. an [M–H]⁻ ion at *m/z* 1119.5176 suggestive of a previously undescribed glycosylation pattern), which guided their targeted isolation [9]. The isolated constituents were subsequently elucidated using nuclear magnetic resonance (NMR) spectroscopy. In addition, their anti-IAV activity was evaluated *in vitro*.

## 2. Material and methods

### 2.1. General experimental procedures

NMR experiments were carried out by a 400 and 600 MHz Bruker AVANCE Neo spectrometer. Optical rotations were obtained using a JASCO DIP-1000. FT-IR data were recorded on a Bruker VERTEX 80 spectrometer. UV spectra were obtained using the UV-1900i UV−Vis spectrophotometer. The experimental ECD spectrum was recorded on a JASCO J-1500 spectropolarimeter. High-resolution electrospray ionization mass spectrometry (HRESIMS) data were obtained on a XEVO G2-XS QT of mass spectrometer and a TripleTOF 5600 plus mass spectrometer. Thin-layer chromatography (TLC) was performed on glass plates precoated with silica gel 60 F254 and RP-18 F254 (20 × 20 cm, 200 µm, 60 Å). Medium-pressure liquid chromatography (MPLC) was conducted using a Biotage Isolera apparatus equipped with a reversed phase C_18_ column (Biotage, Uppsala). Preparative high-performance liquid chromatography (HPLC) was carried out on a Gilson system (PLC 2020) using a Phenomenex Kinetex 5 µm Biphenyl column (250 × 21.2 mm, 5 µm, Phenomenex, Torrance). Analytical HPLC was performed on a Biphenyl C_18_ column (250 × 4.6 mm, 5 µm) equipped with a Shimadzu SPD-M20A photodiode array (PDA) detector.

### 2.2. Plant material

The aerial parts of *P. americana* were collected in 2023 from Daejeon, Korea, and identified by Prof. MinKyun Na (College of Pharmacy, Chungnam National University). A voucher specimen (CNU 20231117) was deposited at the Pharmacognosy Laboratory of the College of Pharmacy, Chungnam National University (Daejeon, Korea).

### 2.3. UPLC–QTOF–MS characterization

UPLC–QTOF–MS analysis was performed in negative ion mode under the following conditions: capillary voltage, 2.5 kV; cone voltage, 40 V; flow rate of desolvation gas (N_2_), 800 l h^−1^; ion source temperature, 150°C; desolvation temperature, 450°C; mass scan range, 100–1500 Da; and scan time, 0.2 s. A sprayer with a reference compound of leucine-enkephalin (*m/z* 554.22615 in negative ion mode) was used as the lock mass. Aliquots (1.0 µl) of each extract sample were injected into an ACQUITY BEH C_18_ column (2.1 × 100 mm, 1.7 µm) and eluted using a chromatographic gradient of two mobile phases (A: 0.1% FA in H_2_O and B: 0.1% FA in MeCN). The linear gradient was optimized as follows: 0.0–15.0 min, 15–50% B; 15.0–20.0 min, 50–100% B; 20.0–25.0 min, 100% B; 25.5–30.0 min, 15% B. The flow rate was 0.4 ml min^−1^, and the temperature of the column oven was maintained at 40°C. The concentration of the extract sample was 3 mg ml^−1^.

### 2.4. Extraction and isolation

The dried aerial parts of *P. americana* (2.8 kg) were extracted with 95% EtOH (36 l) at room temperature for 5 days, followed by two additional reflux extractions (3 h each). The EtOH extract was concentrated *in vacuo* to yield 226.0 g of crude extract. This extract was dissolved in H_2_O (4 l) and successively partitioned with *n*-hexane (Hex) and ethyl acetate (EtOAc). The EtOAc-soluble fraction (84.1 g) was subjected to reversed phase C_18_ silica gel column chromatography (CC) and eluted with a MeOH–H_2_O gradient (10:90–100:0) to afford seven fractions (Fr. 1–7). Fr. 2 (4.62 g) was further separated into four subfractions (Fr. 2.1–2.4) by reversed phase C_18_ silica gel CC (MeCN–H_2_O 20:80). MPLC separation of Fr. 2.1 (2.5 g) afforded four fractions (Fr. 2.11–2.14) using a MeOH–H_2_O gradient (5:95–100:0). Fr. 2.14 (1.69 g) was purified by semi-preparative HPLC (MeCN–H_2_O 13:87) to yield compound **3** (8.8 mg). Fr. 3 (3.1 g) was purified by semi-preparative HPLC (MeCN–H_2_O 25:75) to yield compound **4** (1.3 g). Fr. 4 (9.63 g) was divided into five fractions (Fr. 4.1–4.5) by MPLC (MeOH–H_2_O 40:60–100:0). From Fr. 4.1 (1.84 g), compounds **1** (8.6 mg) and **2** (14.3 mg) were isolated using the same chromatographic conditions as those for Fr. 3. Fr. 4.3 was purified by semi-preparative HPLC (MeCN–H_2_O 28:72) to afford Fr. 4.32 and Fr. 4.33. Fr. 4.32 (277.5 mg) afforded compounds **5** (3.8 mg) and **6** (18.0 mg) by semi-preparative HPLC (MeCN–H_2_O 25:75), while Fr. 4.33 (132.7 mg) yielded compounds **7** (7.0 mg) and **8** (52.3 mg) by semi-preparative HPLC (MeCN–H_2_O 30:70). Fr. 5 (2.14 g) was separated into five fractions (Fr. 5.1–5.5) by MPLC (MeOH–H_2_O 45:55–100:0). Fr. 5.2 (460.2 mg) and Fr. 5.3 (434.3 mg) were purified by semi-preparative HPLC (MeCN–H_2_O 28:72) to give compounds **9** (2.5 mg) and **10** (18.9 mg). Fr. 5.4 (2.6 g) was separated into three fractions (Fr. 5.41–5.43) by semi-preparative HPLC (MeCN–H_2_O 35:65). From Fr. 5.42 (1.0 g), compounds **11** (16.7 mg), **12** (4.5 mg) and **13** (146.8 mg) were isolated using semi-preparative HPLC (MeCN–H_2_O 30:70).

Phytolaccoside P (**1**): amorphous powder; IR (KBr) *ν*_max_ 3406, 1726 and 1640 cm^−1^; ^1^H and ^13^C NMR data, see tables 1 and 2; HRESIMS *m/z* 1165.5288 [M+HCOO]⁻ (calcd for C_54_H_85_O_27_, 1165.5278), 1119.5238 [M–H]⁻ (calcd for C_53_H_83_O_25_, 1119.5223).

**Table 1.** ^1^H and ^13^C NMR data for the aglycones of **1–2** in pyridine-*d*_5_ (*δ* in ppm, *j* in Hz).

no.	1^a^		2^b^	
	*δ*_C_, type	*δ*_H_	*δ*_C_, type	*δ*_H_
1	44.6, CH_2_	1.32, d (13.5) 2.36, d (13.5)	44.3, CH_2_	1.30, m 2.32, d (14.0)
2	71.4, CH	4.76, d (12.6)	70.9, CH	4.69, d (9.7)
3	84.4, CH	4.30, m	84.1, CH	4.21, br s
4	43.2, C	–	43.2, C	–
5	48.9, CH	1.79, m	48.9, CH	1.74, m
6	18.3, CH_2_	1.69, d (11.9) 1.79, m	18.5, CH_2_	1.51, m 1.74, m
7	33.2, CH_2_	1.41, d (10.2) 1.79, m	33.3, CH_2_	1.37, m
8	40.4, C	–	40.4, C	–
9	47.9, CH	1.87, d (11.6)	48.6, CH	1.74, m
10	37.3, C	–	37.3, C	–
11	24.3, CH_2_	2.08, t (13.0)	24.3, CH_2_	2.06, m
12	123.3, CH	5.60, br t	123.5, CH	5.57, br t
13	144.1, C	–	144.1, C	–
14	42.7, C	–	42.7, C	–
15	28.6, CH_2_	1.56, d (13.5) 2.43, dd (13.5, 4.6)	28.6, CH_2_	1.30, m 2.39, m
16	23.8, CH_2_	2.08, t (13.0)	23.9, CH_2_	2.06, m
17	46.9, C	–	46.9, C	–
18	43.5, CH	3.24, dd (13.5, 3.5)	43.6, CH	3.22, d (12.2)
19	42.5, CH_2_	1.79, m 2.25, d (13.0)	42.6, CH_2_	1.74, m 2.23, d (11.5)
20	44.3, C	–	44.5, C	–
21	30.8, CH_2_	1.32, d (13.5) 2.08, t (13.0)	30.9, CH_2_	1.37, m 2.06, m
22	34.3, CH_2_	1.94, d (6.7)	34.4, CH_2_	1.93, d (7.2)
23	65.2, CH_2_	3.73, br d (7.4) 4.41, overlapped	66.4, CH_2_	3.70, d (8.6) 4.31, overlapped
24	15.3, CH_3_	1.37, s	15.1, CH_3_	1.43, s
25	17.9, CH_3_	1.20, s	17.9, CH_3_	1.16, s
26	17.6, CH_3_	1.62, s	17.8, CH_3_	1.56, s
27	26.5, CH_3_	1.26, s	26.5, CH_3_	1.22, s
28	176.4, C	–	176.4, C	–
29	28.6, CH_3_	1.20, s	28.7, CH_3_	1.18, s
30	177.3, C	–	177.3, C	–
COOCH_3_	52.0, CH_3_	3.62, s	52.1, CH_3_	3.60, s

a Spectra was run at 400 MHz (^1^H) and 100 MHz (^13^C).

b Spectra was run at 600 MHz (^1^H) and 150 MHz (^13^C).

**Table 2.** ^1^H and ^13^C NMR data for the sugar units of **1–2** in pyridine-*d*_5_ (*δ* in ppm, *j* in Hz).

no.	1^a^		no.	2^b^	
	*δ*_C_, type	*δ*_H_		*δ*_C_, type	*δ*_H_
3-α-L-arabinopyranosyl			3-β-D-xylopyranosyl		
1′	106.1, CH	5.27, d (6.4)	1′	104.7, CH	5.14, d (6.5)
2′	72.3, CH	4.68, t (8.8)	2′	79.8, CH	4.40, m
3′	83.1, CH	4.30, m	3′	79.2, CH	4.31, overlapped
4′	69.7, CH	4.76, d (12.6)	4′	71.3, CH	4.06, dd (13.3, 7.4)
5′	67.2, CH_2_	3.81, dd (11.6, 1.1) 4.30, m	5′	67.1, CH_2_	3.63, d (10.6)4.23,m
3′-β-D-glucopyranoside			2′-β-D-glucopyranoside		
1″	106.7, CH	5.01, d (6.8)	1″	102.9, CH	5.82, d (7.4)
2″	74.4, CH	4.00, m	2″	79.3, CH	4.31, overlapped
3″	88.3, CH	4.00, m	3″	79.8, CH	4.40, m
4″	71.2, CH	4.41, overlapped	4″	72.5, CH	4.16, t (9.5)
5″	77.5, CH	4.20, overlapped	5″	79.1, CH	4.31, overlapped
6″	62.2, CH_2_	4.41, overlapped	6″	63.3, CH_2_	4.40, m 4.48, d (11.2)
3″-α-L-arabinopyranosyl			2″-α-L-rhamnopyranosyl		
1‴	107.2, CH	5.24, d (7.1)	1‴	103.1, CH	6.31, br d
2‴	73.2, CH	4.52, m	2‴	72.6, CH	4.83, br s
3‴	74.4, CH	4.20, overlapped	3‴	73.0, CH	4.69, d (9.7)
4‴	69.8, CH	4.00, m	4‴	74.6, CH	4.31, overlapped
5‴	66.8, CH_2_	3.54, t (10.4) 4.20, overlapped	5‴	70.4, CH	4.96, d (7.7)
			6‴	19.3, CH_3_	1.80, d (5.9)
28-β-D-glucopyranoside					
1‴′	96.1, CH	6.33, d (8.0)	1‴′	96.2, CH	6.33, br d
2‴′	74.7, CH	4.20, overlapped	2‴′	74.5, CH	4.21, br s
3‴′	79.2, CH	4.30, m	3‴′	78.3, CH	3.90, m
4‴′	69.6, CH	4.30, m	4‴′	71.3, CH	4.40, m
5‴′	79.6, CH	4.00, m	5‴′	80.2, CH	4.00, d (8.3)
6‴′	62.3, CH_2_	4.41, overlapped	6‴′	62.3, CH_2_	4.40, m

a Spectra was run at 400 MHz (^1^H) and 100 MHz (^13^C).

b Spectra was run at 600 MHz (^1^H) and 150 MHz (^13^C).

2β-Hydroxyesculentoside R (**2**): amorphous powder; IR (KBr) *ν*_max_ 3416, 1728 and 1549 cm^−1^; ^1^H and ^13^C NMR data, see tables 1 and 2; HRESIMS *m/z* 1179.5457 [M+HCOO]⁻ (calcd for C_55_H_87_O_27_, 1179.5435), 1133.5399 [M–H]⁻ (calcd for C_54_H_85_O_25_, 1133.5380).

(7*R*,8*S*)-3,5,9,3*′*,9*′*-Pentahydroxy-4*′*,7-epoxy-7*′*-ene-8,5*′*-neolignan (**3**): yellowish amorphous powder; ${\left[\alpha \right]}_{D}^{25}$: −20.0 (*c* = 0.1, CHCl_3_); IR (KBr) *ν*_max_ 3425, 1608, 1446, 1286 and 1018 cm^−1^; UV (MeOH) *λ*_max_ (log *ε*) 276 (0.70), 206 (1.50) nm; CD (MeOH) *λ*_max_*(Δε)* 210 (+0.81) and 296 (−1.30) nm; ^1^H and ^13^C NMR data, see table 3; HRESIMS *m*/*z* 659.2129 [2M–H]⁻ (calcd for C_36_H_35_O_12_, 659.2123), 329.1025 [M–H]⁻ (calcd for C_18_H_17_O_6_, 329.1020), 311.0918 [M–H–H_2_O]⁻ (calcd for C_18_H_15_O_5_, 311.0914) and 299.0915 [M–CH_2_OH]⁻ (calcd for C_17_H_15_O_5_, 299.0914).

**Table 3.** ^1^H (400 MHz) and ^13^C (100 MHz) NMR data for compound **3** in CD_3_OD (*δ* in ppm, *j* in Hz).

	3	
no.	*δ*_C_, type	*δ*_H_
1	135.0, C	–
2	113.9, CH	6.86, d (1.4)
3	146.5, C	–
4	116.2, CH	6.76, br s
5	146.3, C	–
6	118.6, CH	6.77, br s
7	89.0, CH	5.49, d (5.9)
8	55.4, CH	3.48, dd (11.0, 5.9)
9	65.1, CH_2_	3.79, dd (11.0, 5.9) 3.85, dd (11.0, 5.9)
1′	132.2, C	–
2′	115.1, CH	6.83, d (1.4)
3′	142.2, C	–
4′	148.4, C	–
5′	130.3, C	–
6′	115.4, CH	6.89, br s
7′	132.3, CH	6.51, d (15.8)
8′	127.0, CH	6.19, dt (15.8, 6.0)
9′	63.9, CH_2_	4.22, dd (6.0, 1.3)

### 2.5. Acid hydrolysis and sugar analysis of compounds **1−2**

Acid hydrolysis of compounds **1** and **2** was performed according to a previously reported procedure [10]. Compounds **1** and **2** (5.0 mg) were hydrolysed in trifluoroacetic acid (3 ml, 2 M) at 120°C for 3 h and then subjected to concentration under reduced pressure. The residue was dissolved in pyridine (0.1 ml) containing 0.5 mg ml^−1^l-cysteine methyl ester hydrochloride, and the mixture was stirred at 60°C for 1 h. *O*-Tolyl isothiocyanate (2 µl) was added to each mixture, and the resulting mixture was stirred for another hour at 60°C. The reaction mixture was analysed by reversed phase HPLC equipped with a C_18_ column (Thermo-C_18_, 4.6 × 250.0 mm, 5 µm) at 35°C and detected at 250 nm with isocratic elution of 25% MeCN in 50 mM H_3_PO_4_ for 30 min at a flow rate of 0.8 ml min^−1^. Derivatives of standard sugars were prepared using the same method, and their retention times (*t_R_*) were compared with those of the corresponding sugar derivatives from compounds **1** and **2**. The *t_R_* of the standard sugar derivatives were 14.26 min ( d-glucose), 14.64 min (l-glucose), 18.13 min (d-arabinose), 16.39 min (l-arabinose), 14.87 min (d-xylose), 17.03 min (l-xylose), 14.48 min (d-rhamnose) and 21.88 min ( l-rhamnose).

### 2.6. Cell line culture

Human lung adenocarcinoma A549 cell lines (Cat. No. 10185) and Madin–Darby canine kidney (MDCK) cell lines (Cat. No. 10034) were purchased from the Korean Cell Line Bank (KCLB; Seoul, Republic of Korea). A549 cell lines were maintained in Dulbecco's Modified Eagle's Medium (DMEM; Cat. No. SH30243.01, HyClone, Logan, UT, USA) supplemented with 10% fetal bovine serum (FBS; Cat. No. TMS-013-BKR, EMD Millipore, Burlington, MA, USA) and 1% antibiotic–antimycotic solution (100×; Cat. No. 15240062, Thermo Fisher Scientific, Waltham, MA, USA). MDCK cell lines were cultured in Minimum Essential Medium (MEM; HyClone, Logan, UT, USA) supplemented with 10% FBS and 1% antibiotic–antimycotic solution.

### 2.7. Anti-influenza A virus assay

The antiviral activities of the isolated compounds were evaluated against IAV (A/Brisbane/59/2007 [H1N1]) in A549 cells.

For antiviral assays, A549 cells were infected with IAV at a multiplicity of infection (MOI) of 0.1 or 1. After viral adsorption for 2 h, the inoculum was removed, and the cells were washed with phosphate-buffered saline (PBS). The infected cells were then treated with test compounds or crude extract. The positive control (PC), an ethanol extract of *Justicia procumbens* L. (4 µg ml^−1^), was prepared as described previously [11]. Considering the IAV life cycle, 6 hpi was selected to target the active viral RNA synthesis stage, whereas 24 hpi was chosen to evaluate sustained suppression of viral replication.

An initial screening was performed at 10 and 100 µM for selected purified compounds under the above infection conditions, and relative NS1 RNA levels were quantified by qRT-PCR. Total RNA was extracted from the cells, and viral NS1 RNA levels were normalized to 18S rRNA and expressed as a percentage of the virus-infected control. Dose–response experiments for IC_50_ determination were performed using expanded concentration ranges. IC_50_ values were obtained by nonlinear regression (variable-slope model) in GraphPad Prism based on matched assay conditions.

### 2.8. RNA extraction and cDNA synthesis

Total RNA was extracted from the cultured cells using TRIzol® Reagent (Cat. No. 15596018; Thermo Fisher Scientific, Waltham, MA, USA). Briefly, 1 ml of TRIzol was added to each well for cell lysis, followed by the addition of 200 µl of chloroform (Cat. No. C2432; Sigma-Aldrich, St. Louis, MO, USA) in a 5:1 (v/v) ratio. After a 3 min incubation at room temperature, the mixture was centrifuged at 12 000 × *g* for 15 min at 4°C. The aqueous phase was carefully separated from the interphase and organic phase, transferred to a new 1.7 ml tube and mixed with 2-propanol in a 1:1 (v/v) ratio. Following a 10 min incubation at room temperature, the samples were centrifuged at 12 000 × *g* for 10 min at 4°C. The resulting RNA pellet was washed twice with 75% ethanol, dried to remove residual ethanol and then dissolved in diethyl pyrocarbonate (DEPC)-treated water (Cat. No. WR2004-050-00; Biosesang, Seongnam, Republic of Korea). RNA concentration and purity were measured using a NanoDrop™ 2000/2000c spectrophotometer (Thermo Fisher Scientific). Subsequently, cDNA was synthesized using the cDNA Synthesis Master Mix (Cat. No. R5600-500; GenDEPOT, Katy, TX, USA).

### 2.9. Quantitative real-time PCR

The expression levels of viral and host genes were analysed via quantitative real-time polymerase chain reaction (qRT-PCR) using synthesized cDNA. The viral NS1 gene was detected using the following primers: 5′-TCG CGG TAC CTA ACT GAC ATG ACT-3′ (forward) and 5′-CTG GTC CAT TCT GAC ACA AAG AGG-3′ (reverse). For normalization, 18S ribosomal RNA (18S rRNA) was used as an internal control with the primers 5′-CGG CTT TGG TGA CTC TAG AT-3′ (forward) and 5′-GCG ACT ACC ATC GAA AGT TG-3′ (reverse). qRT-PCR was performed using the AccuPower® 2X GreenStar™ qPCR Master Mix (Cat. No. K-6253; Bioneer, Daejeon, Republic of Korea). Relative gene expression levels were calculated using the 2^ΔΔCt^ method:

(2.1)ΔCt=Cttarget gene−Cthousekeeping genes(2.2)ΔΔCt=AverageΔCtnegative control−ΔCttarget sample(2.3)Relative expression level=2−ΔΔCt

All values were expressed as % of the virus-infected control (set to 100%). Statistical significance was analysed by one-way ANOVA followed by Dunnett's post hoc test.

### 2.10. Cytotoxicity assay

The cytotoxic effects of the isolated compounds and crude extract were evaluated in A549 cell lines using the WST-1 assay. A549 cells were seeded in 96-well plates (Cat. No. 30096; SPL Life Sciences, Pocheon, Republic of Korea) at a density of 1 × 10^4^ cells/well in 100 µl of medium and incubated at 37°C in a humidified atmosphere containing 5% CO_2_ for 16 h. Subsequently, the cells were treated with test samples at serial concentrations of 3.125, 6.25, 12.5, 25, 50 and 100 µM (or µg ml^−1^ for the crude extract) for 24 h. After removing the supernatant, 100 µl of solution containing 10% WST-1 reagent (EZ-Cytox; Cat. No. EX-500; DoGenBio, Seoul, Republic of Korea) was added to each well. The plates were incubated for 30 min at 37°C, and the absorbance was measured at 460 nm using a SpectraMax® ABS Plus Microplate Reader (Molecular Devices, San Jose, CA, USA). Cell viability was calculated as a percentage relative to the untreated control, and CC_50_ values were determined where applicable. Statistical significance was analysed compared with the untreated control, and a *p*-value of less than 0.001 was considered highly significant (***p < 0.001). Only concentrations that did not cause significant cytotoxicity were used for antiviral interpretation.

### 2.11. Immunoblot analysis

A549 cell lines were harvested with cold PBS and centrifuged at 6000 rpm for 5 min at 4°C. After the supernatant was completely removed, the cell pellets were lysed using Pierce™ RIPA Buffer (Cat. No. 89901; Thermo Scientific, Rockford, IL, USA) supplemented with Xpert Protease Inhibitor Cocktail Solution (100×) (Cat. No. P3100-005; GenDEPOT, Katy, TX, USA) and Xpert Phosphatase Inhibitor Cocktail Solution (100×) (Cat. No. P3200-010; GenDEPOT, Katy, TX, USA). The lysates were incubated on ice for 30 min with intermittent mixing every 10 min. Following incubation, the samples were centrifuged at 12 000 × *g* for 10 min at 4°C, and the supernatants were collected. Protein concentrations were determined using the BCA Protein Assay Kit (Cat. No. 23227; Thermo Scientific, Rockford, IL, USA) by measuring absorbance at 562 nm. The protein samples were then diluted in Laemmli's 5× Sample Buffer (Cat. No. EBA-1052; Elpis Biotech, Daejeon, Republic of Korea) and heated at 99°C for 5 min.

Equal amounts of protein were separated by sodium dodecyl sulfate–polyacrylamide gel electrophoresis (SDS–PAGE) and transferred onto polyvinylidene difluoride (PVDF) membranes (Cat. No. IPVH00010; Merck Millipore, Burlington, MA, USA) using Transfer Blotting Paper (Cat. No. T0220-50PK; BYLABS, Hanam, Republic of Korea). The membranes were blocked with 5% skim-milk (Cat. No. 232100; BD Biosciences, Sparks, MD, USA) in 1× Tris-buffered saline with Tween-20 (TBS-T) (Cat. No. 0T007; Bio Solution, Suwon, Republic of Korea) for 1 h at room temperature. After washing three times with 1× TBS-T for 10 min each, the membranes were incubated with primary antibodies against IAV NS1 (1:1000; Cat. No. PA5-32243; Invitrogen, Rockford, IL, USA) and β-actin (1:5000; Cat. No. 3700; Cell Signaling Technology, Danvers, MA, USA) in 5% skim-milk at 4°C for 18 h. The membranes were then washed three times with 1× TBS-T and incubated with the appropriate horseradish peroxidase (HRP)-conjugated secondary antibodies: anti-rabbit IgG (Cat. No. 7074; Cell Signaling Technology) for NS1 and anti-mouse IgG (Cat. No. 7076; Cell Signaling Technology) for β-actin for 1 h at room temperature. After washing three times with 1× TBS-T for 10 min each, protein bands were visualized using SuperSignal™ West Pico PLUS Chemiluminescent Substrate (Cat. No. 34580; Thermo Scientific, Rockford, IL, USA) and detected using the ChemiDoc imaging system (SUPERNOVA-Q1800; Centronics, Daejeon, Republic of Korea).

### 2.12. Plaque assay

To evaluate the inhibitory effects of the isolated compounds and crude extract to assess effects on the infectious virus production of viral infection, a plaque reduction assay was performed. MDCK cell lines were seeded in 12-well plates (Cat. No. 30012; SPL Life Sciences, Pocheon, Republic of Korea) at a density of 3 × 10^5^ cells well^−1^ in 1 ml of medium and incubated at 37°C in a humidified atmosphere containing 5% CO_2_ for 16 h. Subsequently, the cells were infected with IAV (A/Brisbane/59/2007 [H1N1]; 5.6 × 10^6^ PFU ml^−1^) at an MOI of 0.0001 and simultaneously treated with the test samples. The infection was carried out by adding 75 µl of the virus suspension and 75 µl of the test compounds at serial concentrations (10, 20, 30, 40, 50, 60, 70, 80, 90 and 100 µM) or crude extract (10–100 µg ml^−1^) in serum-free MEM (Cat. No. SH30024.01; HyClone, Logan, UT, USA) supplemented with TPCK-trypsin (stock 1 mg ml^−1^) diluted 1:4000 (final concentration: 0.25 µg ml^−1^). The plates were incubated for 1 h with shaking every 15 min to facilitate viral adsorption.

Following adsorption, an overlay medium was prepared by mixing 2% low melting temperature agarose (Cat. No. 50084; Lonza, Rockland, ME, USA) and 2× DMEM (DMEM; Cat. No. 12400-024; Gibco, Grand Island, NY, USA) at a 1:1 ratio to achieve a final concentration of 1% agarose, supplemented with TPCK-trypsin (stock 1 mg ml^−1^) diluted 1:2000 (final concentration: 0.5 µg ml^−1^). The mixture (1 ml well^−1^) was added to the infected monolayers and allowed to solidify at room temperature for 30 min. The plates were then incubated at 34°C in a 5% CO_2_ incubator for 48 h. After incubation, the cells were fixed with 4% paraformaldehyde (Cat. No. PC2031-100-00; Biosesang, Seongnam, Republic of Korea), and the agarose overlay was removed using 75% ethanol. The wells were stained with 1 ml of 0.2% crystal violet solution (Cat. No. V5265; Sigma-Aldrich, St. Louis, MO, USA) for 5 min at room temperature. Finally, the stain was washed with distilled water, and the plates were air-dried for 4 h. The antiviral activity was quantified by counting the number of plaques.

### 2.13. Statistical analysis

Statistical analysis was performed using GraphPad Prism (v.11.1.0, GraphPad Software, San Diego, CA, USA). For comparisons involving more than two groups, one-way ANOVA followed by Dunnett's post hoc test was used. For comparisons between two groups, an unpaired two-tailed Student's *t*-test was applied. A *p*-value <0.05 was considered statistically significant (*p < 0.05, **p < 0.01, ***p < 0.001).

## 3. Results and discussion

### 3.1. Structural elucidation

The chemical composition of the aerial parts of *P. americana* was analysed using UPLC–QTOF–MS. Each peak observed in the base peak ion (BPI) chromatogram was tentatively identified based on its retention time (*t_R_*), experimental and theoretical high-resolution mass (HRMS) data, mass error (ppm), molecular formula and key MS^E^ fragment ions, together with comparison with previously reported phytolaccosides from *Phytolacca* species (table 4). In total, 31 triterpenoid saponins were detected. These compounds exhibited characteristic sequential neutral losses of 162, 146 and 132 Da, corresponding to hexose (e.g. glucose [glc]), deoxyhexose (e.g. rhamnose [rham]) and pentose (e.g. arabinose [ara] or xylose [xyl]) moieties, respectively, which are typical fragmentation features of triterpene glycosides. Based on these MS fragmentation patterns, the detected peaks were classified into five aglycone types. Peaks 1, 2, 5, 7, 14, 17, 19, 21, 22, 23, 27 and 31 were tentatively identified as the phytolaccagenin-type, whereas peaks 3, 8, 11, 12, 13, 15, 16, 18, 25, 26, 28 and 30 were characterized as the phytolaccagenic acid-type. In addition, peaks 4 and 24 were attributed to the oleana-9(11),12-diene-28,29-dioic acid-type, peaks 6 and 20 to the jaligonic acid-type and peak 10 to the (3β,4α,20β)-3,23-dihydroxyolean-12-ene-28,29-dioic acid-type. Among them, peaks 5, 10, 12, 15, 16, 22, 24 and 29 exhibited molecular weights and/or glycosylation patterns inconsistent with those of previously reported constituents and were therefore regarded as putative new components. Based on their MS fragmentation patterns, peak 5 was tentatively characterized as a phytolaccagenin-type saponin bearing glycosyl moieties comprising two pentose units and two hexose units. Peak 10 was putatively characterized as a derivative of (3β,4α,20β)-3,23-dihydroxyolean-12-ene-28,29-dioic acid bearing glycosyl moieties comprising one pentose, one deoxyhexose and two hexose units, while peak 12 was interpreted as a phytolaccagenic acid-type saponin bearing glycosyl moieties comprising one deoxyhexose, one pentose and two hexose units. Similarly, peak 15 was tentatively assigned as a phytolaccagenic acid derivative bearing glycosyl moieties comprising two deoxyhexose units, one pentose unit and one hexose unit, whereas peak 16 was proposed to be a phytolaccagenic acid-type saponin bearing two pentose units. Peak 22 was tentatively characterized as a phytolaccagenin-type saponin bearing glycosyl moieties comprising two pentose units and one hexose unit, peak 24 as an oleana-9(11),12-diene-28,29-dioic acid derivative bearing a pentose unit and peak 29 as a phytolaccagenin derivative bearing glycosyl moieties comprising two deoxyhexose units and one pentose unit. Nevertheless, the exact glycosidic linkage patterns—including identity of the sugar residues, sugar–sugar connectivity and linkage positions—could not be determined and require further confirmation by NMR analysis and/or hydrolysis-based approaches. Guided by the MS-based profiling results (table 4), targeted unreported triterpene saponins were subsequently isolated, and their structures were elucidated by comprehensive spectroscopic analyses, including HRMS, IR and NMR (figure 1).

**Table 4.** UPLC–QTOF–MS–based tentative identification of triterpenoid saponins in *Phytolacca americana*.

peak no.	ESI MS *t_R_* (min)	observed mass (*m/z*)	calculated mass (*m/z*)	error (ppm)	molecular formula	key MS^E^ fragment ions (*m/z*)	identification	references
1	5.266	1033.4825 [M+HCOO]⁻	1033.4856	−3.0	C_48_H_76_O_21_	987.4768 [M–H]⁻ 825.4256 [M–162–H]⁻ 663.3721 [M–162–162–H]⁻ 531.3323 [M–162–162–132–H]⁻	phytolaccasaponin B (esculentoside H)	[12,13]
2	5.813	1179.5370 [M+HCOO]⁻	1179.5435	−5.5	C_54_H_86_O_25_	1133.5625 [M–H]⁻ 971.5074 [M–162–H]⁻ 825.4235 [M–162–146–H]⁻ 663.3759 [M–162–146–162–H]⁻ 531.3317 [M–162–146–162–132–H]⁻	2β-hydroxyesculentoside R	[14]
3	5.920	1017.4855 [M+HCOO]⁻	1017.4906	−5.0	C_48_H_76_O_20_	971.4808 [M–H]⁻ 809.4308 [M–162–H]⁻ 647.3370 [M–162–162–H]⁻ 515.3338 [M–162–162–132–H]⁻	esculentoside L	[15]
4	6.006	869.4130 [M+HCOO]⁻	869.4171	−4.7	C_42_H_64_O_16_	823.4103 [M–H]⁻ 661.3574 [M–162–H]⁻ 529.3110 [M–162–132–H]⁻	3-*O*-[β-D-glucopyranosyl(1–4)-β-D-xylopyranosyl]-28-β-glucopyranosyl-30-methyloleanate-9(11),12-dien-2,3,23-trihydroxyl-28-oic acid	[16]
5	6.090	1119.5176 [M–H]⁻	1119.5223	−4.2	C_53_H_83_O_25_	1165.5525 [M+HCOO]⁻ 957.4664 [M–162–H]⁻ 825.4255 [M–162–132–H]⁻ 663.3733 [M–162–132–162–H]⁻ 531.3338 [M–162–132–162–132–H]⁻	phytolaccoside P	NF
6	6.254	857.4137 [M+HCOO]⁻	857.4171	−4.0	C_41_H_64_O_16_	811.4106 [M–H]⁻ 517.3166 [M–294(162+132)–H]⁻	esculentoside F	[17]
7	6.552	825.4271 [M–H]⁻	825.4273	−0.2	C_42_H_66_O_16_	871.4443 [M+HCOO]⁻ 663.3766 [M–162–H]⁻ 531.3338 [M–162–132–H]⁻	esculentoside S	[18]
8	6.709	1163.5427 [M+HCOO]⁻	1163.5486	−5.1	C_54_H_86_O_24_	1117.5382 [M–H]⁻ 955.4874 [M–162–H]⁻ 809.4304 [M–162–146–H]⁻ 647.3782 [M–162–146–162–H]⁻ 515.3370 [M–162–146–162–132–H]⁻	esculentoside R	[12]
9	6.908	695.3632 [M+HCOO]⁻	695.3643	−1.6	C_35_H_54_O_11_	649.3597 [M–H]⁻ 517.3177 [M–132–H]⁻	phytolaccoside G	[19]
10	7.093	1149.5286 [M+HCOO]⁻	1149.5329	−3.7	C_53_H_84_O_24_	1103.5236 [M–H]⁻ 941.4728 [M–162–H]⁻ 809.4314 [M–162–132–H]⁻ 663.3743 [M–162–132–146–H]⁻ 501.3200 [M–162–132–146–162–H]⁻		NF
11	7.129	1017.4870 [M+HCOO]⁻	1017.4906	−3.5	C_48_H_76_O_20_	971.4828 [M–H]⁻ 809.4313 [M–162–H]⁻ 647.3781 [M–162–162–H]⁻ 515.3365 [M–162–162–132–H]⁻	phytolaccagenic acid 3-*O*-[β-D-glucopyranosyl-(1′′→3′)-α-L-arabinopyranosyl]-28-*O*-β-D-glucopyranoside	[20]
12	7.434	1163.5455 [M+HCOO]⁻	1163.5486	−2.7	C_54_H_86_O_24_	1117.5383 [M–H]⁻ 955.4882 [M–162–H]⁻ 809.4313 [M–162–146–H]⁻ 677.3538 [M–162–146–132–H]⁻ 515.3400 [M–162–146–132–162–H]⁻		NF
13	7.662	855.4349 [M+HCOO]⁻	855.4378	−3.4	C_42_H_66_O_15_	809.4294 [M–H]⁻ 647.3793 [M–162–H]⁻ 515.3348 [M–162–132–H]⁻	phytolaccoside D	[21]
14	7.882	739.3895 [M+HCOO]⁻	739.3905	−1.4	C_37_H_58_O_12_	693.3830 [M–H]⁻ 531.3326 [M–162–H]⁻	esculentoside D	[22]
15	8.074	1147.5471 [M+HCOO]⁻	1147.5536	−5.7	C_54_H_86_O_23_	1101.5417 [M–H]⁻ 939.4935 [M–162–H]⁻ 515.2983 [M–162–424(146+146+132)–H]⁻		NF
16		825.4238 [M+HCOO]⁻	825.4273	−4.2	C_41_H_64_O_14_	779.4191 [M–H]⁻ 515.2983 [M–264(132+132)–H]⁻		NF
17	8.138	1033.4807 [M+HCOO]⁻	1033.4856	−4.7	C_48_H_76_O_21_	987.4765 [M–H]⁻ 825.4252 [M–162–H]⁻ 663.3681 [M–162–162–H]⁻ 531.3183 [M–162–162–132–H]⁻	phytolaccasaponin B (esculentoside H) isomer	
18	8.316	971.4824 [M–H]⁻	971.4852	−2.9	C_48_H_76_O_20_	1017.4849 [M+HCOO]⁻ 515.3011 [M–456(162+162+132)]⁻	esculentoside L isomer	
19	8.529	825.4272 [M–H]⁻	825.4273	−0.1	C_42_H_66_O_16_	871.4377 [M+HCOO]⁻ 663.3743 [M–162–H]⁻ 531.3320 [M–162–132–H]⁻	esculentoside A (phytolaccoside E)	[22,23]
20	8.714	563.3210 [M+HCOO]⁻	563.3220	−1.8	C_30_H_46_O_7_	517.3174 [M–H]⁻	jaligonic acid (desmethylphytoloccagenin)	[24]
21	8.778	825.4244 [M–H]⁻	825.4273	−3.5	C_42_H_66_O_16_	871.4281 [M+HCOO]⁻ 663.3713 [M–162–H]⁻ 531.3278 [M–162–132–H]⁻	esculentoside S/esculentoside A (phytolaccoside E) isomer	
22	8.891	957.4667 [M–H]⁻	957.4695	−2.9	C_47_H_74_O_20_	1003.4692 [M+HCOO]⁻ 825.4241 [M–132–H]⁻ 663.3730 [M–132–162–H]⁻ 531.3278 [M–132–162–132–H]⁻		NF
23	8.991	739.3884 [M+HCOO]⁻	739.3905	−2.8	C_37_H_58_O_12_	693.3839 [M–H]⁻ 531.3312 [M–162–H]⁻	28-*O*-β-D-glucopyranosyl phytolaccagenin	[25]
24	9.034	707.3635 [M+HCOO]⁻	707.3643	−1.1	C_36_H_54_O_11_	661.3587 [M–H]⁻ 529.3161 [M–132–H]⁻		NF
25	9.098	1001.4942 [M+HCOO]⁻	1001.4957	−1.5	C_48_H_76_O_19_	955.4889 [M–H]⁻ 809.4310 [M–146–H]⁻ 647.3796 [M–146–162–H]⁻ 515.3377 [M–146–162–132–H]⁻	phytolaccoside F	[26]
26	9.155	855.4355 [M+HCOO]⁻	855.4378	−2.7	C_42_H_66_O_15_	809.4307 [M–H]⁻ 647.3792 [M–162–H]⁻ 515.3369 [M–162–132–H]⁻	esculentoside T	[27]
27	9.681	1327.7518 [2M–H]⁻	1327.7567	−3.7	C_36_H_56_O_11_	709.3938 [M+HCOO]⁻ 663.3819 [M–H]⁻ 531.3325 [M–132–H]⁻	esculentoside B (phytolaccoside B)	[28]
28	9.958	855.4353 [M+HCOO]⁻	855.4378	−2.9	C_42_H_66_O_15_	809.4303 [M–H]⁻ 647.3762 [M–162–H]⁻ 515.3406 [M–162–132–H]⁻	phytolaccoside D/esculentoside T isomer	
29	10.350	955.4877 [M–H]⁻	955.4903	−2.7	C_48_H_76_O_19_	1001.4912 [M+HCOO]⁻ 809.4301 [M–146–H]⁻ 663.3710 [M–146–146–H]⁻ 531.3241 [M–146–146–132–H]⁻		NF
30	10.719	647.3790 [M–H]⁻	647.3795	−0.8	C_36_H_56_O_10_	693.3830 [M+HCOO]⁻ 515.3332 [M–132–H]⁻	phytolaccoside A	[21]
31	11.530	577.3378 [M+HCOO]⁻	577.3377	0.2	C_31_H_48_O_7_	531.3329 [M–H]⁻	phytolaccagenin	[28]

***t_R_***: retention time; NF: not found.

**Figure 1.**
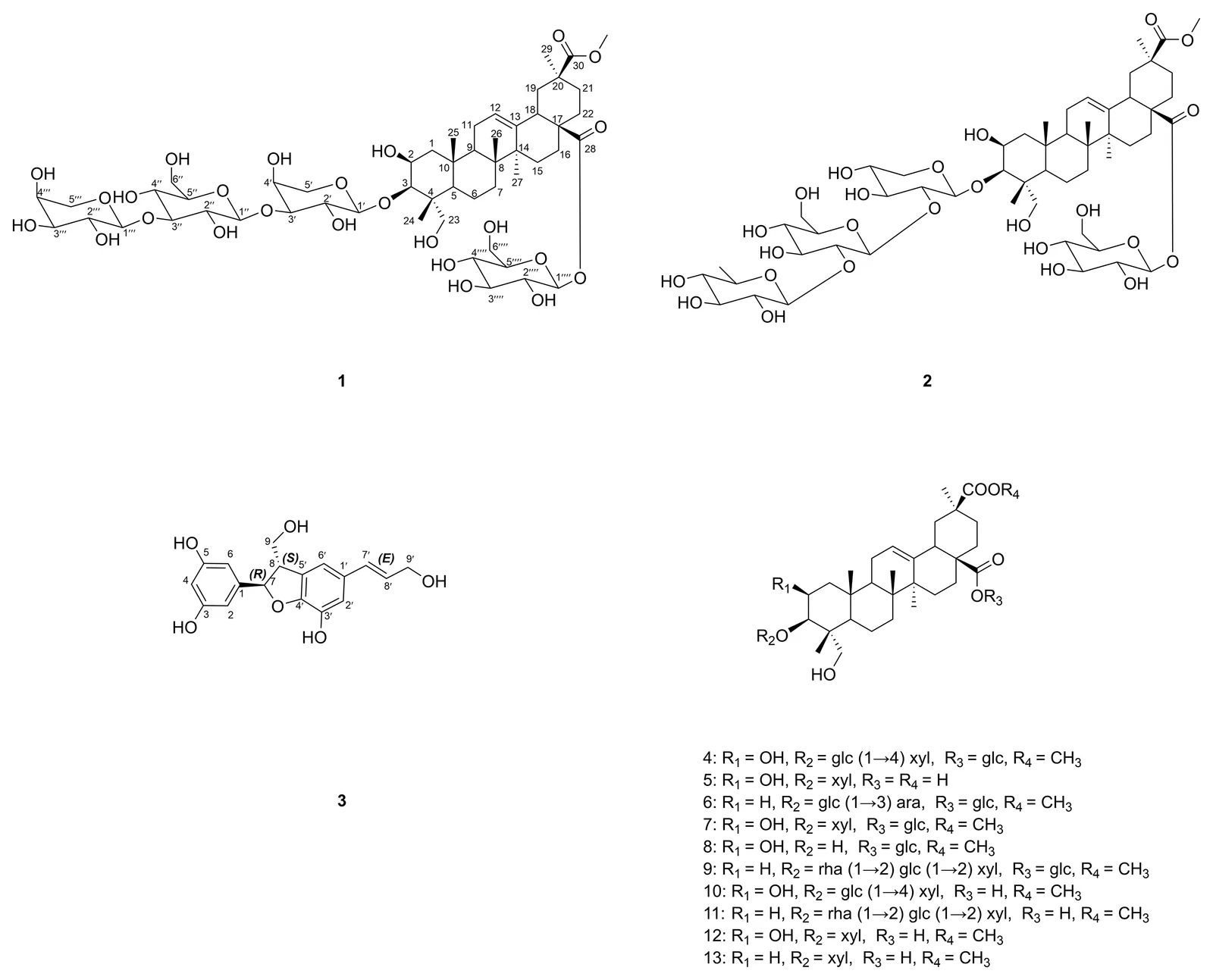
Structures of compounds **1**–**13** isolated from the aerial parts of *Phytolacca americana*.

Compound **1** was obtained as an amorphous powder. Its negative HRESIMS (UPLC–QTOF–MS) spectrum showed a formate adduct ion at *m/z* 1165.5288 [M+HCOO]⁻ (calcd for C_54_H_85_O_27_, 1165.5278). The deprotonated ion at *m/z* 1119.5238 [M–H]⁻ (calcd for C_53_H_83_O_25_, 1119.5223) was also observed, which was consistent with the molecular formula C_53_H_84_O_25_ (supplementary material, figure S1). Furthermore, a series of fragment ions at *m/z* 957.4680 (−162 Da, hexose) and 825.4263 (−132 Da, additional pentose) were observed. Subsequent fragment ions at *m/z* 663.3720 [M–162–132–162–H]⁻ and *m/z* 531.3335 [M–162–132–162–132–H]⁻ were detected, corresponding to step-wise losses of two hexose and two pentose units (total neutral loss: 588 Da). The key fragment ion at *m/z* 531.3335 is consistent with a phytolaccagenin-type aglycone (figure 2) [2]. The IR spectrum indicated absorption bands for hydroxyl (3406 cm^−1^), carbonyl (1726 cm^−1^) and olefinic (1640 cm^−1^) groups. The ^1^H-NMR spectroscopic data (tables 1and 2) of **1** showed four anomeric proton signals at *δ*_H_ 6.33 (d, *J* = 8.0 Hz), 5.27 (d, *J* = 6.4 Hz), 5.24 (d, *J* = 7.1 Hz) and 5.01 (d, *J* = 6.8 Hz), an olefinic proton at *δ*_H_ 5.60 (br t), a methoxy signal at *δ*_H_ 3.62 (s), as well as five methyl groups at *δ*_H_ 1.62, 1.37, 1.26, 1.20 and 1.20. The ^13^C-NMR spectroscopic data (tables 1 and 2) exhibited resonances for two carbonyl carbons (*δ*_C_ 177.3 and 176.4), two olefinic carbon signals (*δ*_C_ 144.1 and 123.3), four anomeric carbons (*δ*_C_ 107.2, 106.7, 106.1 and 96.1), one methoxy carbon (*δ*_C_ 52.0) and five methyl groups (*δ*_C_ 28.6, 26.5, 17.9, 17.6 and 15.3). Characteristic carbon signals at *δ*_C_ 144.1 and 123.3 were assigned to C-13 and C-12, respectively, together with C-18 at *δ*_C_ 43.5 and *δ*_H_ 3.24 (dd, *J* = 13.5, 3.5 Hz), confirming an olean-12-ene triterpenoid skeleton. In contrast to the typical oleanane triterpenes bearing methyl groups at C-23, C-28 and C-30, the aglycones in this study exhibited oxidative modifications characteristic of phytolaccagenin-type triterpenes. In particular, the carbonyl carbon resonated at *δ*_C_ 177.3 and showed a key HMBC cross-peak with the methoxy protons at *δ*_H_ 3.62 (OC*H*_3_), establishing a methyl ester at C-30. The stereochemistry of **1** was determined by NOESY correlations (figure 3) between H-2/H-9 and H-3/H-9, which indicated that H-2 and H-3 are α-oriented, while correlations between H-5/CH_3_-27, H-9/CH_3_-27 and H-9/CH_3_-29 corroborated the axial arrangement of these methyl groups. Conversely, sequential NOESY correlations among CH_3_-24/CH_3_-25, CH_3_-24/CH_3_-26 and CH_3_-25/CH_3_-26 confirmed their β-orientations. A detailed analysis of the two-dimensional NMR data (^1^H–^1^H COSY, TOCSY, HSQC, DEPT-135, HMBC, NOESY) enabled the identification of the aglycone of **1** as a 2β,3β-hydroxylated phytolaccagenin-type triterpene. In the HSQC spectrum, four anomeric carbons were observed at *δ*_C_ 107.2, 106.7, 106.1 and 96.1, with corresponding anomeric protons at *δ*_H_ 5.24, 5.01, 5.27 and 6.33, respectively. The units with anomeric protons at *δ*_H_ 6.33 (d, *J* = 8.0 Hz) and 5.01 (d, *J* = 6.8 Hz) corresponded to two hexoses with their methylene carbons at *δ*_C_ 62.3 and 62.2, identified as β-glucopyranose. The remaining anomeric protons at *δ*_H_ 5.27 (d, *J* = 6.4 Hz) and 5.24 (d, *J* = 7.1 Hz), along with the characteristic inverted CH_2_ signals at *δ*_C_ 67.2 and 66.8 in the DEPT-135, were consistent with the reported spectra of α-arabinopyranosides recorded in pyridine-*d*_5_ [29]. In the HMBC data (figure 4), the anomeric proton at *δ*_H_ 5.27 (Ara-H-1′) showed a key correlation with the oxymethine carbon at *δ*_C_ 84.4 (C-3), confirming that the inner arabinose was directly attached to C-3 of the aglycone. A further HMBC cross-peak from *δ*_H_ 5.01 (Glc-H-1″) to *δ*_C_ 83.1 (Ara-C-3′) indicated that the glucose residue was linked to C-3′ of arabinose. In addition, the correlation from *δ*_H_ 5.24 (Ara-H-1‴) to *δ*_C_ 88.3 (Glc-C-3″) established the terminal arabinose attached to C-3″ of the glucose unit, and together these correlations defined the trisaccharide unit attached at C-3 as α-arabinopyranose-(1‴→3″)-β-glucopyranose-(1″→3′)-α-arabinopyranose. Similarly, the HMBC cross-peak between *δ*_H_ 6.33 (Glc-H-1‴ ′) and the ester carbonyl carbon at *δ*_C_ 176.4 (C-28) demonstrated that the second glucosyl residue was attached at C-28 of phytolaccagenin. Acid hydrolysis of **1**, followed by chemical derivatization, afforded l-arabinose and d-glucose, which were identified by HPLC analysis through comparison with authentic standards (supplementary material, figure S31). Thus, the structure of **1** was determined as 3-*O*-[α-l-arabinopyranosyl-(1‴→3″)-β-d-glucopyranosyl-(1″→3′)-α-l-arabinopyranosyl]-28-O-β-d-glucopyranosyl phytolaccagenin and was named phytolaccoside P.

**Figure 2.**
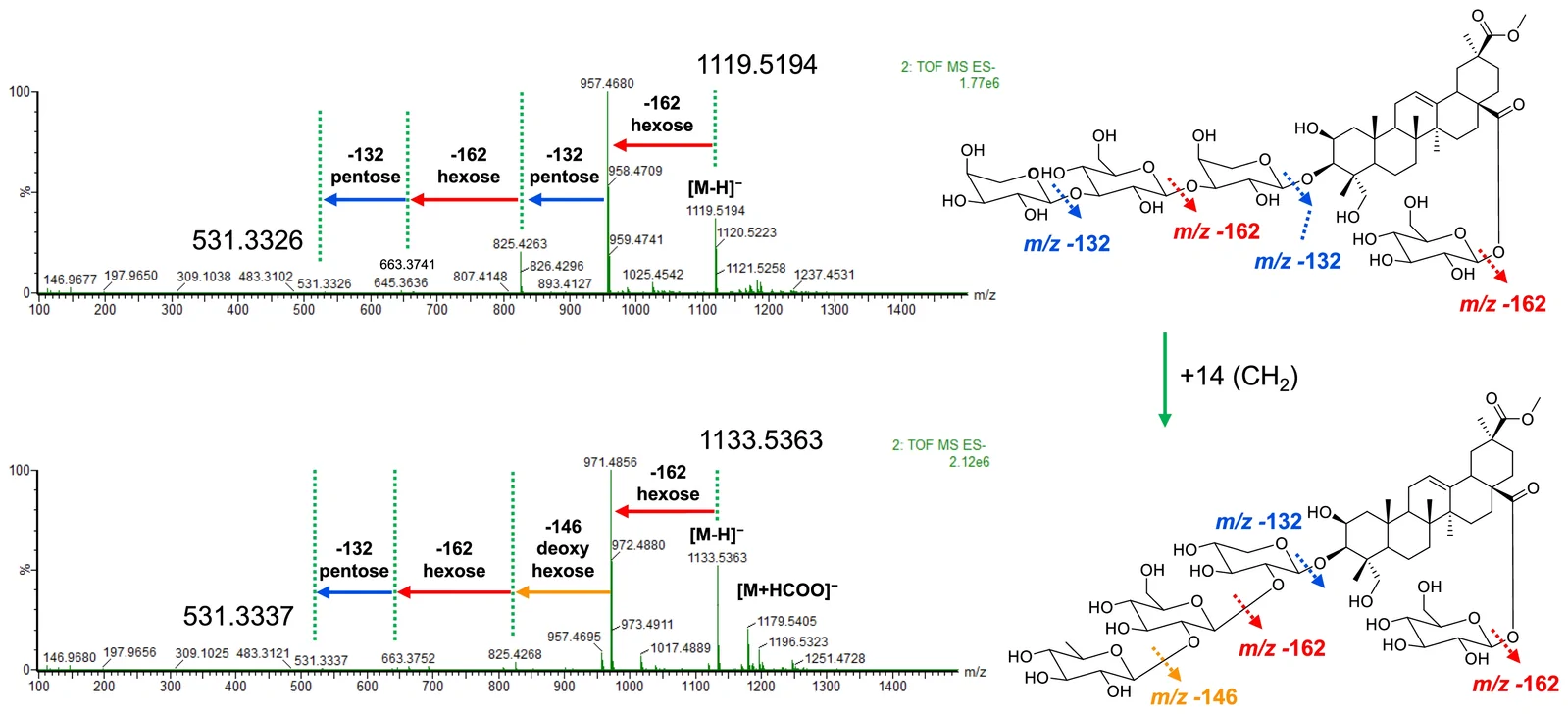
UPLC–QTOF–MS/MS spectra of compounds **1**–**2**.

**Figure 3.**
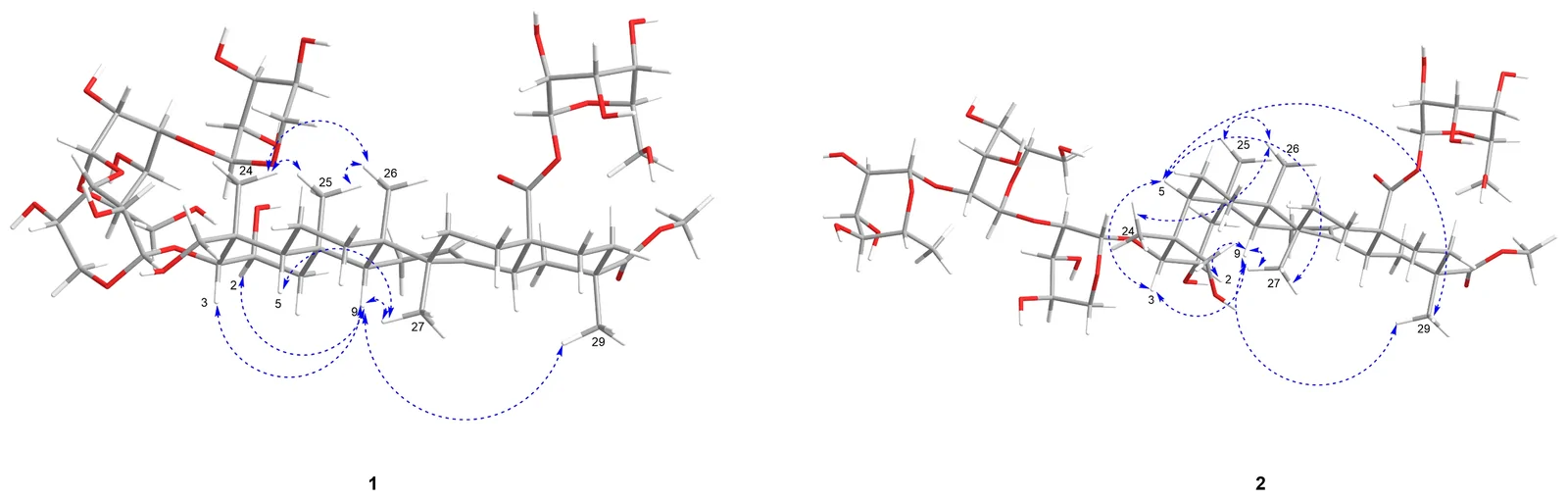
The key ^1^H–^1^H NOESY (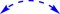) correlations of compounds **1**–**2**.

**Figure 4.**
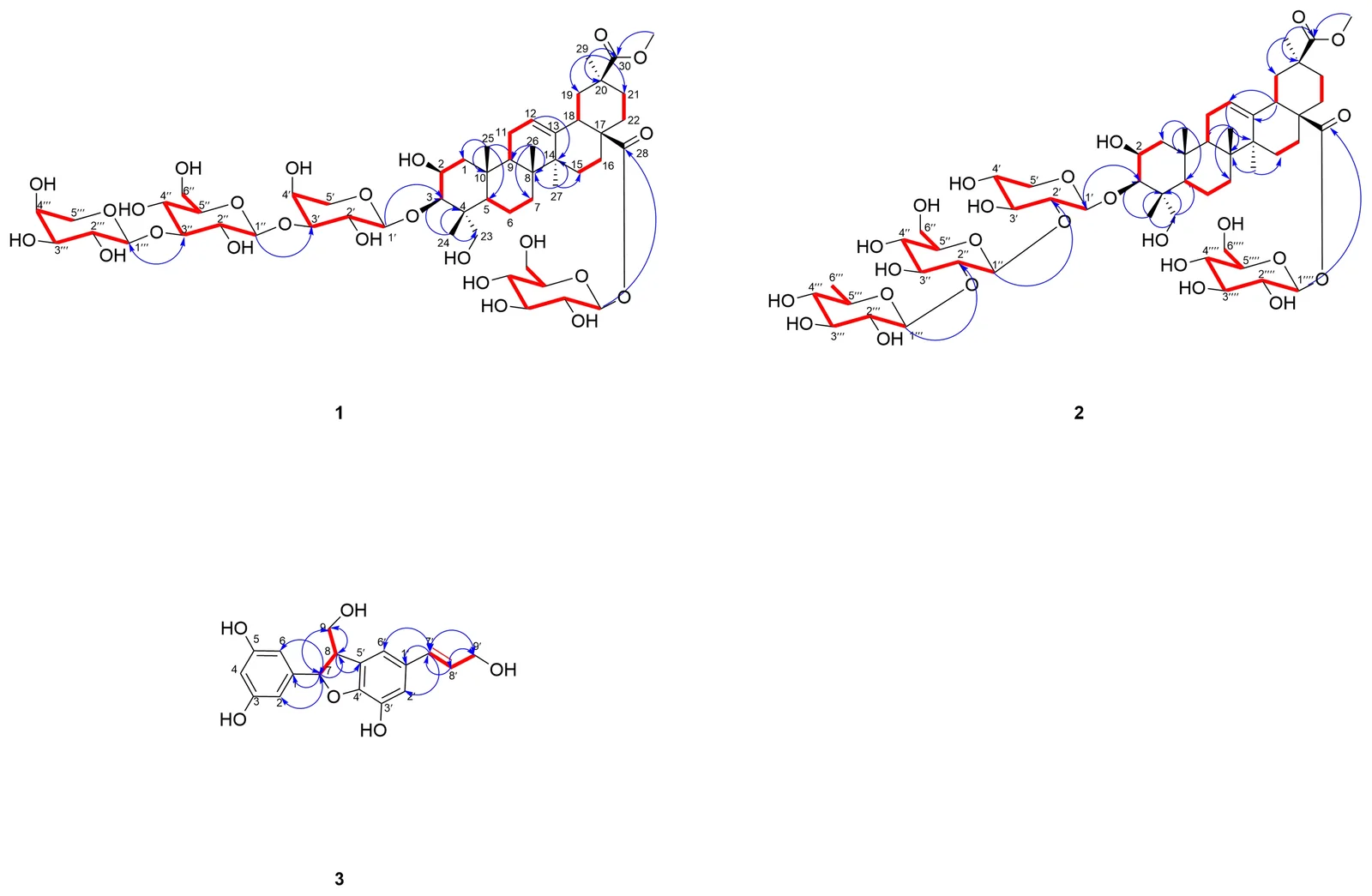
The key ^1^H–^1^H COSY (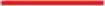) and key ^1^H–^13^C HMBC (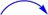) correlations of compounds **1**–**3**.

Compound **2** was obtained as an amorphous powder. The negative HRESIMS of **2** showed a formate adduct ion at *m/z* 1179.5457 [M+HCOO]⁻ (calcd for C_55_H_87_O_27_, 1179.5435), indicating that **2** contains one additional CH_2_ unit (14 Da) compared with compound **1**. The deprotonated ion at *m/z* 1133.5399 [M–H]⁻ (calcd for C_54_H_85_O_25_, 1133.5380) was also observed, in agreement with the molecular formula C_54_H_86_O_25_ (supplementary material, figure S11). A fragment ion at *m/z* 971.4856, corresponding to the loss of a hexose unit (162 Da), was observed. Subsequent fragmentation produced an ion at *m/z* 825.4268 [M–162–146–H]⁻, corresponding to the loss of a deoxyhexose unit (146 Da). Further loss of a second hexose produced the ion at *m/z* 663.3752 [M–162–146–162–H]⁻, followed by a pentose loss (132 Da) yielding *m/z* 531.3337 [M–162–146–162–132–H]⁻. Finally, two hexoses, one deoxyhexose and one pentose residue, were sequentially lost, resulting in the observation of the characteristic ion at *m/z* 531.3334 of a phytolaccagenin-type aglycone (figure 2) [2]. The IR spectrum confirmed the presence of hydroxyl (3416 cm^−1^), carbonyl (1728 cm^−1^) and olefinic (1549 cm^−1^) groups. Analysis of its HRESIMS, ^1^H- and ^13^C-NMR spectroscopic data (tables 1 and 2) revealed that compound **2** exhibited an aglycone identical to that of **1**, except for the glycosidic units. The anomeric protons at *δ*_H_ 6.33 (br d) and 5.82 (d, *J* = 7.4 Hz), together with the methylene carbons at *δ*_C_ 62.3 and 63.3, corresponded to two β-glucopyranose, as in **1**. By contrast, the anomeric signal at *δ*_H_ 5.14 (d, *J* = 6.5 Hz) and *δ*_C_ 104.7, along with a characteristic pentose C-5 resonance at *δ*_C_ 67.1, suggested a β-xylopyranose residue [30]. The remaining anomeric proton at *δ*_H_ 6.31 (br d), together with a methyl doublet at *δ*_H_ 1.80 (d, *J* = 5.9 Hz) and *δ*_C_ 19.3, indicated an α-rhamnopyranose moiety [31]. HMBC correlations (figure 4) from *δ*_H_ 5.14 (Xyl-H-1′) to *δ*_C_ 84.1 (C-3) indicated that the xylose residue was directly attached to C-3 of the aglycone. Further correlations from *δ*_H_ 5.82 (Glc-H-1″) to *δ*_C_ 79.8 (Xyl-C-2′) and from *δ*_H_ 6.31 (Rha-H-1‴) to *δ*_C_ 79.3 (Glc-C-2″) revealed that the trisaccharide unit attached at C-3 was α*-*rhamnopyranose-(1‴→2″)-β-glucopyranose-(1″→2′)-β-xylopyranose. The anomeric signal at *δ*_H_ 6.33 (Glc-H-1‴ ′) with its carbon at *δ*_C_ 96.2 showed an HMBC correlation with the ester carbonyl at *δ*_C_ 176.4 (C-28), confirming a 28-*O*-β-glucopyranose moiety, the same as **1**. Acid hydrolysis and subsequent derivatization released d-glucose, l-rhamnose and d-xylose, as identified by HPLC comparison with authentic standards (supplementary material, figure S32). Analysis of ^1^H–^1^H COSY, TOCSY, HSQC, HMBC and DEPT-135 experiments, together with NOESY correlations, revealed that **2** possesses the same 2β,3β-dihydroxylated oleanane-type aglycone as **1** (figure 3). The molecular structure of **2** has been tentatively identified in Phytolacca *acinosa* Roxb. using the HPLC-ESI-QTOF MS technique [14]. However, the isolation of this compound and its structure elucidation are reported for the first time in this study. Therefore, these spectroscopic data established the structure of **2** as 3-*O*-[α-l-rhamnopyranosyl-(1‴→2″)-β-d-glucopyranosyl-(1″→2′)-β-d-xylopyranosyl]-28-*O*-β-d-glucopyranosyl phytolaccagenin, and the common name 2β-hydroxyesculentoside R was assigned.

Compound **3** was obtained as a yellowish amorphous solid, with a specific rotation of ${\left[\alpha \right]}_{D}^{25}$: −20.0 (*c* = 0.1, CHCl_3_). The molecular formula was determined as C_18_H_18_O_6_ by HRESIMS with *m/z* 659.2129 [2M–H]^−^ (calcd 659.2123). The IR absorptions at 3425, 1608, 1446 and 1286 cm^−1^ indicated the presence of hydroxyl groups, double-bond and benzene-ring groups, respectively. The ^1^H NMR data (table 3) displayed the presence of five aromatic proton singlets at *δ*_H_ 6.89 (br s, H-6′), 6.86 (d, *J* = 1.4 Hz, H-2), 6.83 (d, *J* = 1.4 Hz, H-2′), 6.77 (br s, H-6) and 6.76 (br s, H-4), indicating the presence of 1,3,5-trisubstituted and 1′,3′,4′,5′-tetrasubstituted aromatic rings, two olefinic protons at *δ*_H_ 6.51 (d, *J* = 15.8 Hz, H-7′) and 6.19 (dt, *J* = 15.8, 6.0 Hz, H-8′), one oxygenated methine *δ*_H_ 5.49 (d, *J* = 5.9 Hz, H-7), two oxygenated methylenes *δ*_H_ 4.22 (dd, *J* = 6.0, 1.3 Hz, H-9′), 3.85 (dd, *J* = 11.0, 5.9 Hz, H-9b) and 3.79 (dd, *J* = 11.0, 5.9 Hz, H-9a) and one methine *δ*_H_ 3.48 (dd, *J* = 11.0, 5.9 Hz, H-8). The ^13^C NMR spectrum (table 3) revealed 18 carbon peaks, including 12 aromatic carbons for two aromatic rings at *δ*_C_ 146.5 (C-3), 146.3 (C-5), 135.0 (C-1), 118.6 (C-6), 116.2 (C-4) and 113.9 (C-2); and 148.4 (C-4′), 142.2 (C-3′), 132.2 (C-1′), 130.3 (C-5′), 115.1 (C-2′) and 115.4 (C-6′). By comprehensive analysis of the HSQC, HMBC, ^1^H–^1^H COSY and NOESY spectra (figure 4; supplementary material, figure S28), the proton and carbon resonances of compound **3** were found to be similar to those of previously reported dihydrobenzofuran neolignans, indicating that compound **3** possesses a dihydrobenzofuran neolignan skeleton bearing hydroxyl groups at C-3, C-5 and C-3′, which was further supported by key HMBC correlations from H-2, H-4, H-6, H-2′ and H-6′ [32]. The coupling constant between H-7′ and H-8′ (*J* = 15.8 Hz) permitted the assignment of the orientation of the olefinic bond between C-7′ and C-8′ as *E*, which was further supported by the NOESY correlation of H-7′ with H-9′. Furthermore, the typical coupling constant between H-7 and H-8 (*J* = 5.9 Hz) and the chemical shift *δ*_C_ 55.4 (C-8) suggested the orientation of H-7 and H-8 to be *trans*, which was further supported by the NOESY correlations of H-7 with H-9 [33–35]. The absolute configuration of **3** was elucidated as 7*R* and 8*S* based on its CD spectrum, which showed Cotton effects at 210 nm (+0.81) and 296 nm (−1.30), respectively (supplementary material, figure S27), by comparison with the reported CD spectrum of hierochin C [36]. Consequently, compound **3** was established as (7*R*,8*S*)-3,5,9,3′,9′-pentahydroxy-4′,7-epoxy-7′-ene-8,5′-neolignan.

Ten known compounds (**4**–**13**) were obtained and identified as esculentoside H (**4**) [12], phytolaccoside G (**5**) [19], 3-*O*-β-d-glucopyranosyl-(1→3)-*O*-α-l-arabinopyranosyl phytolaccagenic acid-28-*O*-β-d-glucopyranosyl ester (**6**) [29], esculentoside S (**7**) [18], 28-*O*-β-d-glucopyranosyl phytolaccagenin (**8**) [25], esculentoside R (**9**) [12], phytolaccoside E (**10**) [23], phytolaccoside F (**11**) [26], phytolaccoside B (**12**) [3] and phytolaccoside A (**13**) [21], by comparing their spectroscopic data with those reported in the literature. The chemical structures of the known compounds **4**‒**13** are shown in figure 1.

### 3.2. Anti-influenza A virus activities

Previous studies have reported the antiviral potential of *P. americana* leaf extracts [8]. Accordingly, the present study evaluated the anti-IAV activity of the crude extract and isolated compounds using complementary *in vitro* assays. A549 cells were infected with H1N1 (A/Brisbane/59/2007) at an MOI of 1, and viral NS1 RNA levels were evaluated by endpoint PCR and RT–qPCR. In the initial screening at 10 µM, the isolated compounds generally reduced NS1 RNA levels at 6 hpi. By contrast, the crude extract at 10 µg ml^−1^ did not show a significant inhibitory effect (supplementary material, figures S33 and S34). The antiviral effects were subsequently evaluated at 100 µM for the isolated compounds and 100 µg ml^−1^ for the crude extract. At 6 hpi, most tested samples markedly reduced NS1 RNA levels (figure 5A). At 24 hpi, compounds **9**–**13** significantly reduced NS1 RNA levels, while compound **11** exhibited the most pronounced inhibitory effect among the isolated compounds (figure 5B).

**Figure 5.**
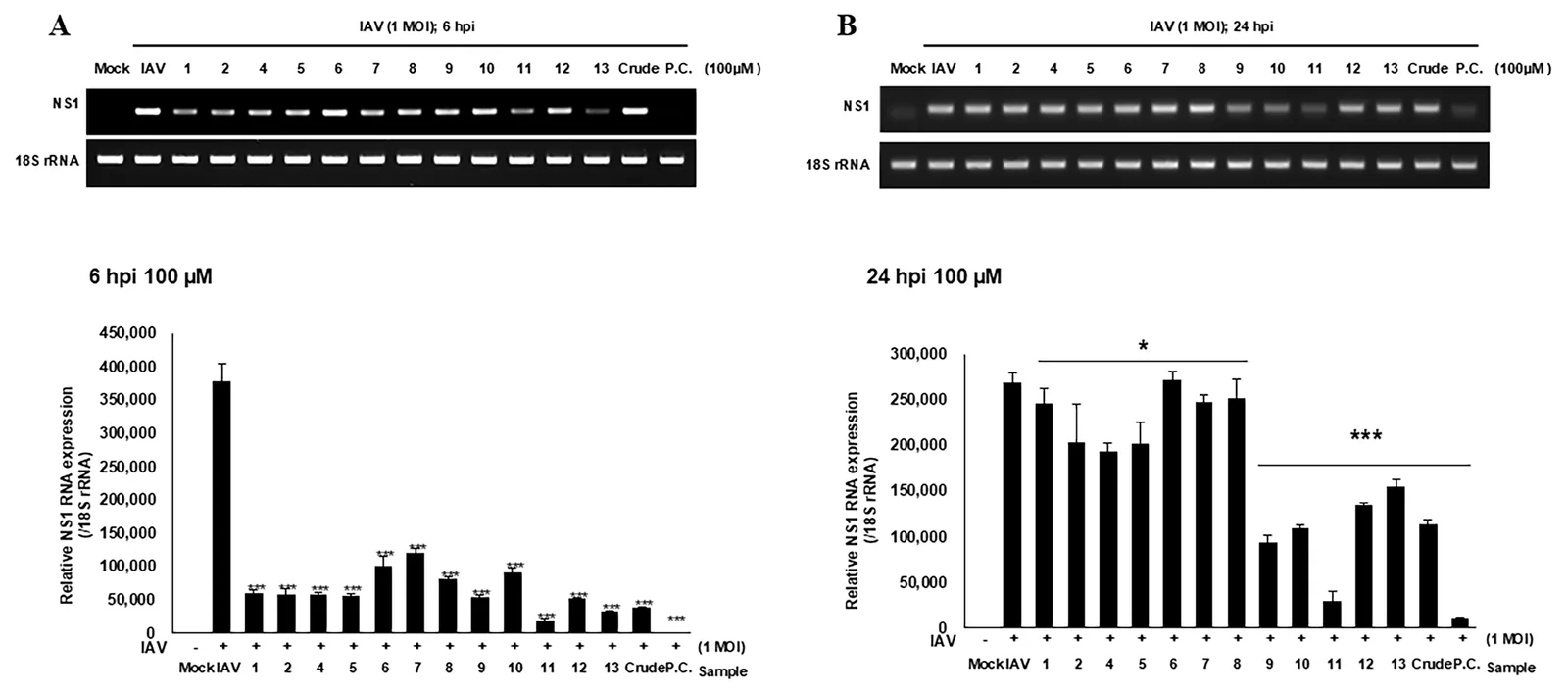
Effects of isolated compounds and crude extract on NS1 mRNA expression in IAV-infected A549 cells. A549 cells were infected with IAV at an MOI of 1 and treated with isolated compounds (100 µM), crude extract (100 µg ml^−1^) or positive control (PC; 4 µg ml^−1^). NS1 mRNA expression was evaluated by endpoint PCR and RT–qPCR at (A) 6 hpi and (B) 24 hpi. *p < 0.05, and ***p < 0.001 vs. the virus-infected control.

Based on its sustained inhibitory effect on NS1 expression at 24 hpi, compound **11** (figure 6A) was selected for further evaluation. Its antiviral effect was subsequently examined at the protein level in IAV-infected A549 cells at an MOI of 0.1. At 24 hpi, intracellular NS1 protein levels decreased with increasing concentrations of compound **11** (figure 6B). Similarly, NP levels in the culture supernatants decreased in a concentration-dependent manner (figure 6C). The crude extract also reduced NS1 and NP levels under the same conditions (supplementary material, figure S35). Cytotoxicity evaluation showed that compound **11** did not markedly affect A549 cell viability at concentrations up to 100 µM (CC_50_ > 100 µM) (figure 6D). To further determine whether the observed inhibition was associated with reduced production of infectious virus, compound **11** was evaluated using a plaque reduction assay in MDCK cells infected with IAV at an MOI of 0.0001. Compound **11** inhibited plaque formation in a concentration-dependent manner, with an IC_50_ value of 57.98 ± 0.26 µM (figure 6E). Plaque reduction assay was also performed for compounds **12** and **13**, which showed IC_50_ values of 97.26 ± 0.28 and 53.41 ± 0.58 µM, respectively (supplementary material, figure S36). The crude extract also showed plaque reduction activity, with IC_50_ values of 8.32 ± 0.01 and 4.63 ± 0.03 µg ml^−1^ in the initial and subsequent dose–response assays, respectively (supplementary material, figure S36). However, the crude extract exhibited cytotoxicity at higher concentrations, with a CC_50_ value of 68.1 ± 0.77 µg ml^−1^ (supplementary material, figure S37). Collectively, these results indicate that compound **11** consistently inhibited viral markers at both the RNA and protein levels and reduced infectious virus production.

**Figure 6.**
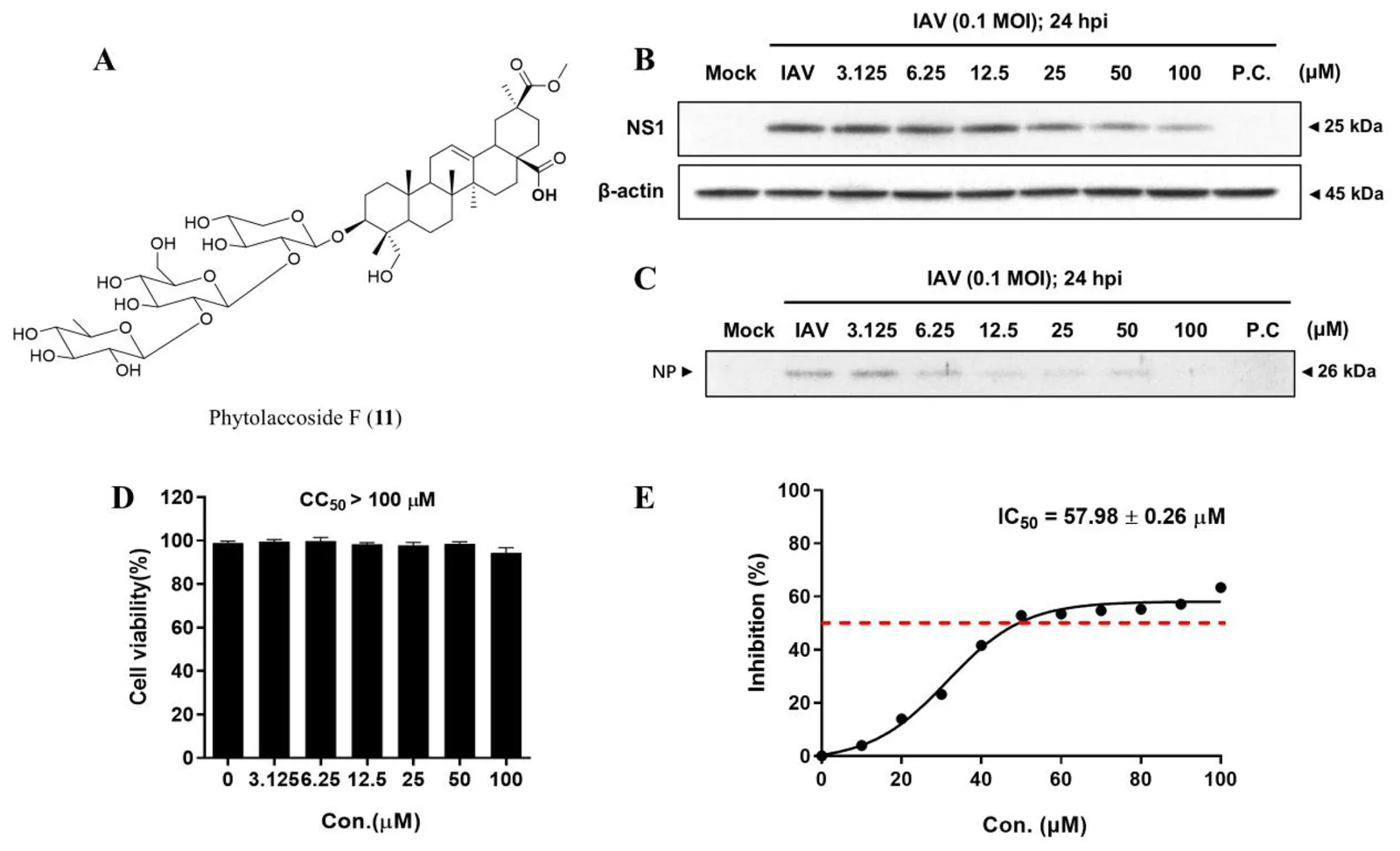
Anti-influenza A virus activity of phytolaccoside F (**11**). (A) Chemical structure of phytolaccoside F (**11**). (B) Intracellular NS1 protein levels and (C) NP levels in culture supernatants of IAV-infected A549 cells treated with compound **11**. (D) Cytotoxicity of compound **11** in A549 cells. (E) Plaque reduction activity of compound **11** in IAV-infected MDCK cells. PC, positive control.

All tested saponins share an oleanane-type triterpenoid scaffold but differ in C-2 hydroxylation, C-3 and C-28 glycosylation. Comparison of compounds **9** and **11**, which share the same C-3 trisaccharide and differ primarily in C-28 glycosylation, showed a stronger reduction in NS1 RNA levels for compound **11** at 24 hpi, suggesting that the absence of a C-28 glucose moiety may be associated with antiviral activity. The potential influence of C-2 hydroxylation was examined by comparing compounds **12** and **13**, which share a C-3 xylosyl moiety and lack C-28 glycosylation but differ in the presence of a C-2 hydroxyl group. Compound **13** showed greater activity than compound **12** in the plaque reduction assay (IC_50_ = 53.41 ± 0.58 and 97.26 ± 0.28 µM, respectively) (supplementary material, figure S36), suggesting that the absence of a C-2 hydroxyl group may contribute to enhanced antiviral activity. The C-3 glycoside structure may also contribute to activity. Notably, compound **11**, bearing a trisaccharide at C-3, showed consistent inhibitory effects across RT–qPCR, plaque reduction and protein-level assays. This observation is consistent with previous reports suggesting that trisaccharide moieties may contribute to the anti-influenza activity of triterpene saponins [5–7]. Nevertheless, given the limited number of structurally related compounds examined, these observations should be regarded as preliminary, and additional analogues will be required to clarify the individual contributions of C-2 hydroxylation, C-3 glycoside structure and C-28 glycosylation to antiviral activity.

## 4. Conclusions

In conclusion, comprehensive UPLC–QTOF–MS analysis of the extract of *P. americana* led to the tentative annotation of 31 saponin-related peaks, demonstrating the chemical complexity of structurally diverse triterpenoid saponins in this species. Guided by MS fragmentation patterns, previously unreported constituents were targeted for isolation, resulting in the structural elucidation of one new oleanane-type triterpenoid saponin (**1**), the complete structural determination of **2** for the first time, one new neolignan (**3**) and 10 known metabolites (**4**–**13**). Using the plaque assay, compound **11** was identified as the most reproducible antiviral constituent. Structurally, it lacks glycosylation at C-28 and bears a trisaccharide moiety at C-3. Preliminary SAR analysis suggests that these structural characteristics may contribute to its antiviral activity; however, further validation through comparison with additional analogues is required. Collectively, these findings expand the phytochemical profile of *P. americana* and suggest phytolaccagenin-type triterpenoid saponins as promising antiviral scaffolds for further investigation.

## Supplementary Material



## Data Availability

The data supporting the findings of this study are available within the article and its supplementary material [37].
