# Supplementary material for: Triterpenoid saponins from the aerial parts of Phytolacca americana and their anti-influenza A virus activity
Source: R Soc Open Sci. 2026 Nov 4;13(11):262034. doi: 10.1098/rsos.262034 (PMC13730658; doi:10.1098/rsos.262034)
Supplement: Supplementary file 1 [file rsos.262034.zip › rsos.262034/rsos262034_si_001.docx]

**Supporting Information**

**Triterpenoid saponins from the aerial parts of *Phytolacca americana* and their anti-influenza A virus activity**

Shuo Tian,^a,1^ Jisu Park,^a,1^ You Jin Kim,^b^ Sainan Li,^a^ Wonkyun Ronny Im,^b,c^ Sung Ho Jeon,^b,^* MinKyun Na,^a,^*

^a^ *College of Pharmacy, Chungnam National University, Daejeon 34134, Republic of Korea*

^b^ *Department of Life Science and Multidisciplinary Genome Institute, Hallym University, Chuncheon, 24252, Republic of Korea*

^c^ *Hallym AI-Biohealth R&BD Center, Medical Bio-Convergence Research Institute, Hallym University, Chuncheon 24252, Republic of Korea*

Journal: *Royal Society Open Science*
Article DOI: 10.1098/rsos.262034

* Corresponding author.

E-mail addresses: sjeon@hallym.ac.kr (Sung Ho Jeon)

mkna@cnu.ac.kr (MinKyun Na)

^1^ These authors have contributed equally to this work.

**Contents**

[Figure S1. HRESIMS data of **1**	4](#_Toc238716606)

[Figure S2. ^1^H-NMR spectrum of **1** (400 MHz, Pyridine-*d*_5_)	5](#_Toc238716607)

[Figure S3. ^13^C-NMR spectrum of **1** (100 MHz, Pyridine-*d*_5_)	6](#_Toc238716608)

[Figure S4. DEPT-135 spectrum of **1** (Pyridine-*d*_5_)	7](#_Toc238716609)

[Figure S5. COSY spectrum of **1** (Pyridine-*d*_5_)	8](#_Toc238716610)

[Figure S6. HSQC spectrum of **1** (Pyridine-*d*_5_)	9](#_Toc238716611)

[Figure S7. HMBC spectrum of **1** (Pyridine-*d*_5_)	10](#_Toc238716612)

[Figure S8. NOESY spectrum of **1** (Pyridine-*d*_5_)	11](#_Toc238716613)

[Figure S9. TOCSY spectrum of **1** (Pyridine-*d*_5_)	12](#_Toc238716614)

[Figure S10. IR spectrum of **1** (KBr)	13](#_Toc238716615)

[Figure S11. HRESIMS data of **2**	14](#_Toc238716616)

[Figure S12. ^1^H-NMR spectrum of **2** (600 MHz, Pyridine-*d*_5_)	15](#_Toc238716617)

[Figure S13. ^13^C-NMR spectrum of **2** (150 MHz, Pyridine-*d*_5_)	16](#_Toc238716618)

[Figure S14. DEPT-135 spectrum of **2** (Pyridine-*d*_5_)	17](#_Toc238716619)

[Figure S15. COSY spectrum of **2** (Pyridine-*d*_5_)	18](#_Toc238716620)

[Figure S16. HSQC spectrum of **2** (Pyridine-*d*_5_)	19](#_Toc238716621)

[Figure S17. HMBC spectrum of **2** (Pyridine-*d*_5_)	20](#_Toc238716622)

[Figure S18. NOESY spectrum of **2** (Pyridine-*d*_5_)	21](#_Toc238716623)

[Figure S19. TOCSY spectrum of **2** (Pyridine-*d*_5_)	22](#_Toc238716624)

[Figure S20. IR spectrum of **2** (KBr)	23](#_Toc238716625)

[Figure S21. HRESIMS data of **3**	24](#_Toc238716626)

[Figure S22. ^1^H-NMR spectrum of **3** (400 MHz, CD_3_OD)	25](#_Toc238716627)

[Figure S23. ^13^C-NMR spectrum of **3** (100 MHz, CD_3_OD)	26](#_Toc238716628)

[Figure S24. COSY spectrum of **3** (CD_3_OD)	27](#_Toc238716629)

[Figure S25. HMQC spectrum of **3** (CD_3_OD)	28](#_Toc238716630)

[Figure S26. HMBC spectrum of **3** (CD_3_OD)	29](#_Toc238716631)

[Figure S27. NOESY spectrum of **3** (CD_3_OD)	30](#_Toc238716632)

[Figure S28. The key ^1^H-^1^H NOESY (](#_Toc238716633)[) correlations of **3**	31](#_Toc238716633)

[Figure S29. CD spectrum of **3** (CD_3_OD)	32](#_Toc238716634)

[Figure S30. IR spectrum of **3** (KBr)	33](#_Toc238716635)

[Figure S31. HPLC chromatogram of acid-hydrolyzed compound **1** and standard sugar	34](#_Toc238716636)

[Figure S32. HPLC chromatogram of acid-hydrolyzed compound **2** and standard sugar	35](#_Toc238716637)

[Figure S33. RT–qPCR analysis of NS1 mRNA expression at 6 hpi in A549 cells infected with IAV at an MOI of 1	36](#_Toc238716638)

[Figure S34. Representative end-point PCR analysis of NS1 mRNA at 6 and 24 hpi in A549 cells infected with IAV at an MOI of 1	37](#_Toc238716639)

[Figure S35. Additional immunoblot analyses of the antiviral effects of the crude extract	38](#_Toc238716640)

[Figure S36. Additional dose–response evaluation of compounds **11**–**13** and the crude extract by plaque reduction assay	39](#_Toc238716641)

[Figure S37. Cytotoxicity of compounds **12** and **13** and the crude extract in A549 cells	40](#_Toc238716642)

# Figure S1. HRESIMS data of 1

# Figure S2. ^1^H-NMR spectrum of 1 (400 MHz, Pyridine-*d*_5_)

# Figure S3. ^13^C-NMR spectrum of 1 (100 MHz, Pyridine-*d*_5_)

# Figure S4. DEPT-135 spectrum of 1 (Pyridine-*d*_5_)

# Figure S5. COSY spectrum of 1 (Pyridine-*d*_5_)

# Figure S6. HSQC spectrum of 1 (Pyridine-*d*_5_)

# Figure S7. HMBC spectrum of 1 (Pyridine-*d*_5_)

# Figure S8. NOESY spectrum of 1 (Pyridine-*d*_5_)

# Figure S9. TOCSY spectrum of 1 (Pyridine-*d*_5_)

# Figure S10. IR spectrum of 1 (KBr)

# Figure S11. HRESIMS data of 2

# Figure S12. ^1^H-NMR spectrum of 2 (600 MHz, Pyridine-*d*_5_)

# Figure S13. ^13^C-NMR spectrum of 2 (150 MHz, Pyridine-*d*_5_)

# Figure S14. DEPT-135 spectrum of 2 (Pyridine-*d*_5_)

# Figure S15. COSY spectrum of 2 (Pyridine-*d*_5_)

# Figure S16. HSQC spectrum of 2 (Pyridine-*d*_5_)

# Figure S17. HMBC spectrum of 2 (Pyridine-*d*_5_)

# Figure S18. NOESY spectrum of 2 (Pyridine-*d*_5_)

# Figure S19. TOCSY spectrum of 2 (Pyridine-*d*_5_)

# Figure S20. IR spectrum of 2 (KBr)

# Figure S21. HRESIMS data of 3

# Figure S22. ^1^H-NMR spectrum of 3 (400 MHz, CD_3_OD)

# Figure S23. ^13^C-NMR spectrum of 3 (100 MHz, CD_3_OD)

# Figure S24. COSY spectrum of 3 (CD_3_OD)

# Figure S25. HMQC spectrum of 3 (CD_3_OD)

# Figure S26. HMBC spectrum of 3 (CD_3_OD)

# Figure S27. NOESY spectrum of 3 (CD_3_OD)

# Figure S28. The key ^1^H-^1^H NOESY () correlations of 3

# Figure S29. CD spectrum of 3 (CD_3_OD)

# Figure S30. IR spectrum of 3 (KBr)

# Figure S31. HPLC chromatogram of acid-hydrolyzed compound 1 and standard sugar

# Figure S32. HPLC chromatogram of acid-hydrolyzed compound 2 and standard sugar

# Figure S33. RT–qPCR analysis of NS1 mRNA expression at 6 hpi in A549 cells infected with IAV at an MOI of 1

A549 cells were infected with IAV at an MOI of 1 and treated with test compounds (10 μM), crude extract (10 μg/mL), or positive control. NS1 mRNA levels were quantified by RT–qPCR at 6 hpi, normalized to 18S rRNA, and expressed relative to the virus-infected control. Data are presented as mean ± SEM (n = 3). ns, not significant; **p* < 0.05 and ****p* < 0.001 vs the virus-infected control.

# Figure S34. Representative end-point PCR analysis of NS1 mRNA at 6 and 24 hpi in A549 cells infected with IAV at an MOI of 1

A549 cells were infected with IAV at an MOI of 1 and treated with test compounds (10 μM), crude extract (10 μg/mL), or positive control. NS1 mRNA was analyzed by end-point PCR at 6 or 24 hpi, with 18S rRNA used as an internal control. Mock indicates non-infected cells, and P.C. indicates the positive control.

# Figure S35. Additional immunoblot analyses of the antiviral effects of the crude extract

Corresponding immunoblot analyses for the crude extract. A549 cells were infected with IAV at an MOI of 0.1 and treated with crude extract (3.125–100 μg/mL). Samples were collected at 24 hpi. β-Actin was used as a loading control for the NS1 immunoblot analysis. P.C. indicates the positive control.

# Figure S36. Additional dose–response evaluation of compounds 11–13 and the crude extract by plaque reduction assay

(A) Refined dose–response evaluation of compound **11**. (B) Dose–response evaluation of compound **12**. (C) Refined dose–response evaluation of compound **13**. (D, E) Initial and refined dose–response evaluations of the crude extract, respectively. MDCK cells were infected with IAV at an MOI of 0.0001 and treated with the indicated concentrations of compounds 11–13 (μM) or crude extract (μg/mL). Percent inhibition was calculated relative to the virus-only control, and IC_50_ values were determined by nonlinear regression of the dose–response curves (variable-slope model) in GraphPad Prism.

# Figure S37. Cytotoxicity of compounds 12 and 13 and the crude extract in A549 cells

A549 cells were treated with compounds **12** and **13** (3.125–100 μM) or crude extract (3.125–100 μg/mL) for 24 h. Cell viability was determined using the WST-1 assay and expressed as a percentage of the untreated control (set to 100%). Data are presented as mean ± SEM (n = 3). CC_50_ values are indicated in the panels.
