# Supplementary material for: Triterpenoid saponins from the aerial parts of Phytolacca americana and their anti-influenza A virus activity
Source: R Soc Open Sci. 2026 Nov 4;13(11):262034. doi: 10.1098/rsos.262034 (PMC13730658; doi:10.1098/rsos.262034)
Supplement: Supplementary file 1 [file rsos.262034.zip › rsos.262034/rsos262034_si_002.pdf]

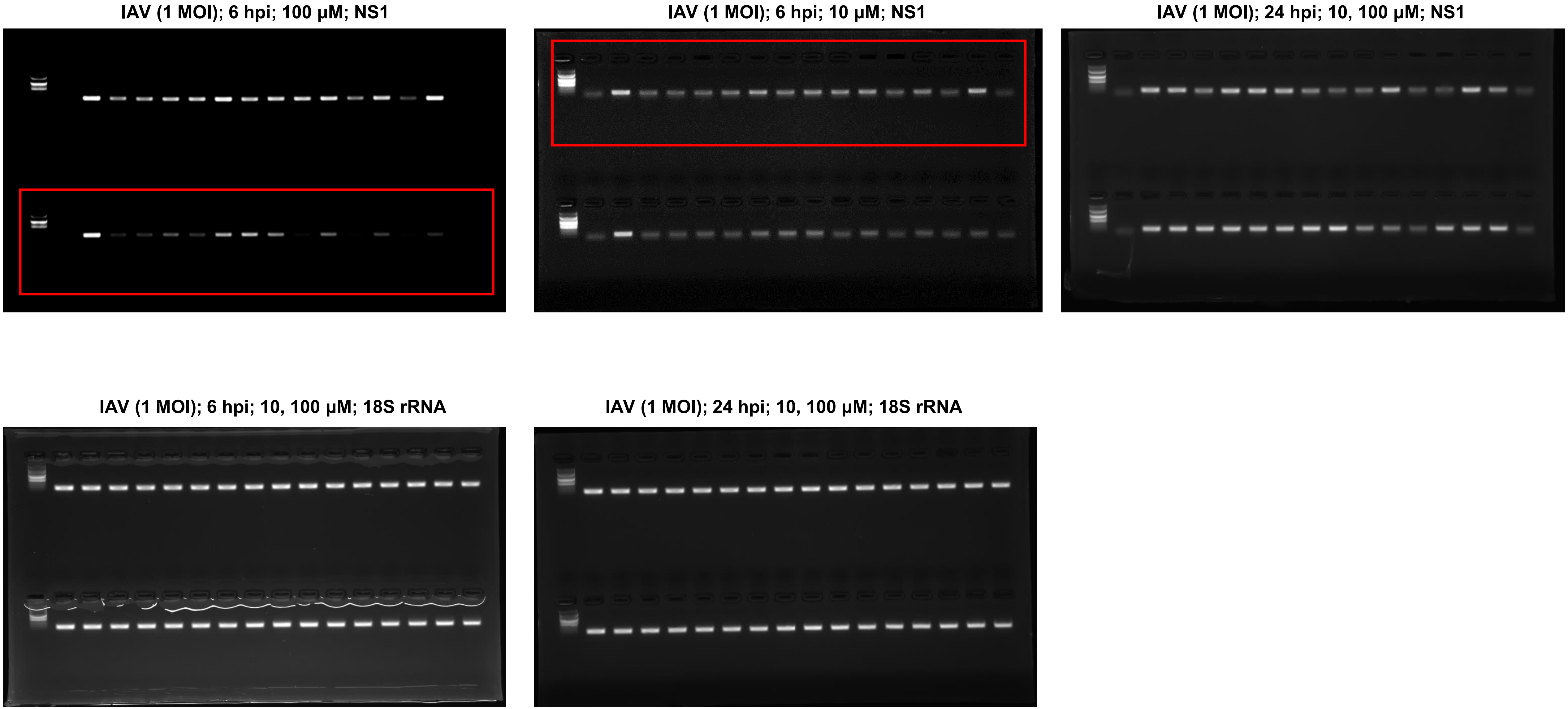

Fig 5. Original gel images for end-point PCR analysis of NS1 and 18S rRNA.

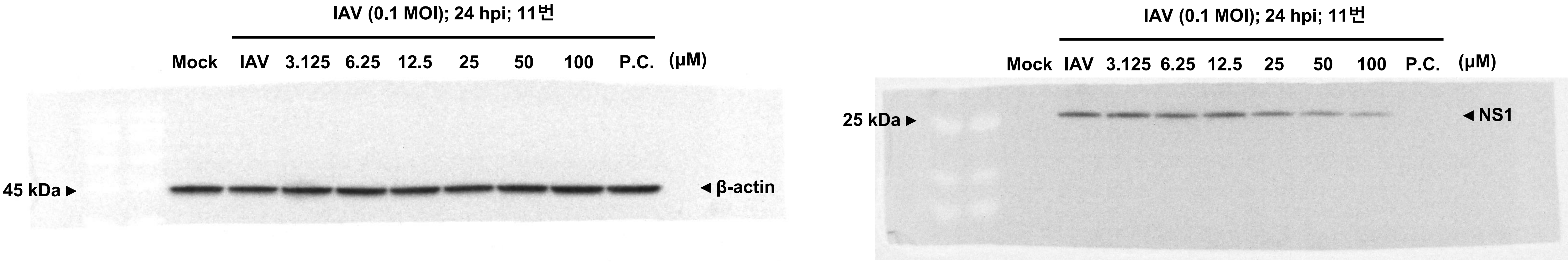

Fig 6B. Original immunoblot images for NS1 and β-actin under 0.1 MOI infection.

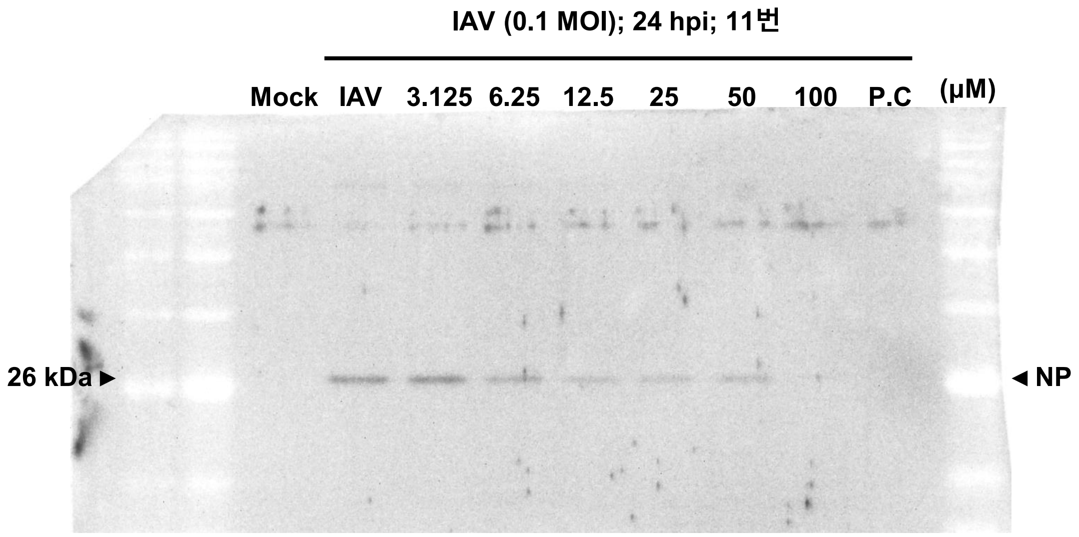

Fig 6C. Original immunoblot images for NP in culture supernatants under 0.1 MOI infection.

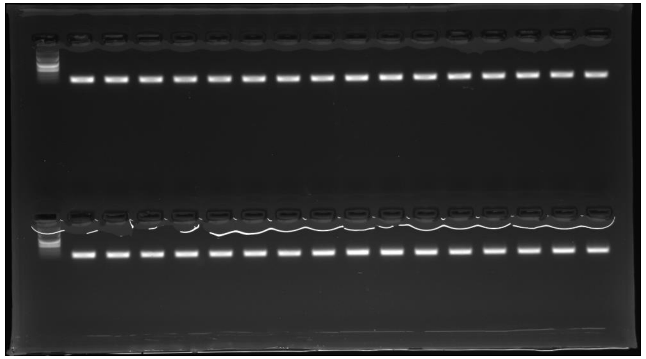

Fig S34. 10(top) uM_18S rRNA 6 h

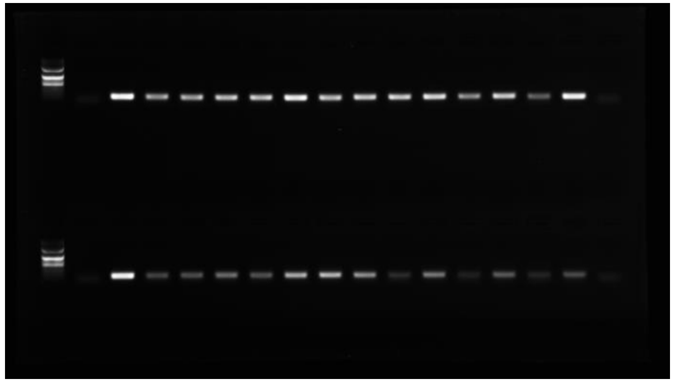

Fig S34. 10(top) uM_NS1 6 h

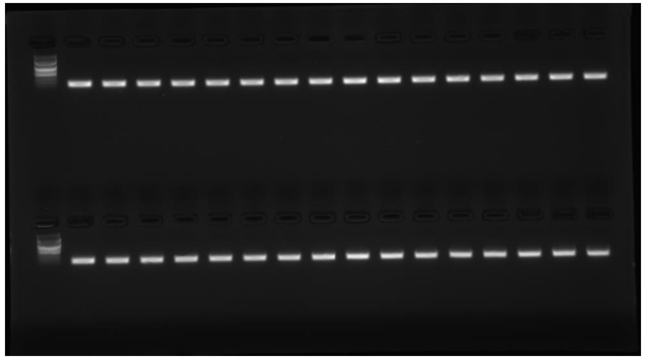

Fig S34. 10(top), 100(bottom) uM_18S rRNA 24 h

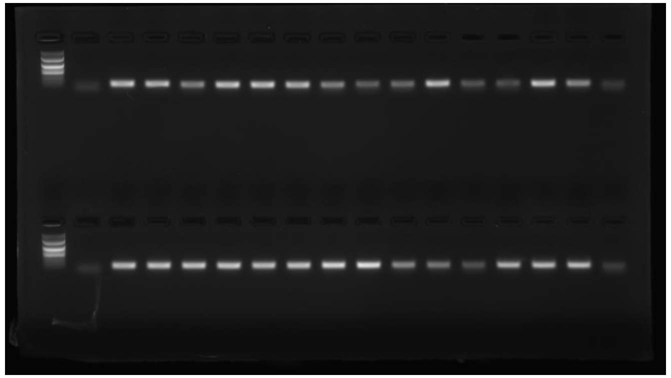

Fig S34. 10(up), 100(bottom) uM_NS1 24 h

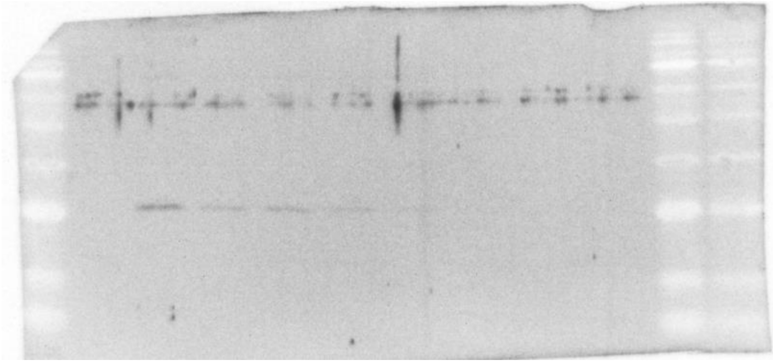

Fig S35. Crude_NP

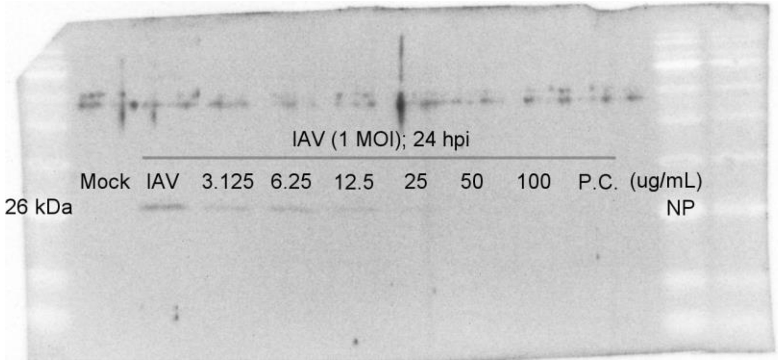

Fig S35. Crude_NP-1

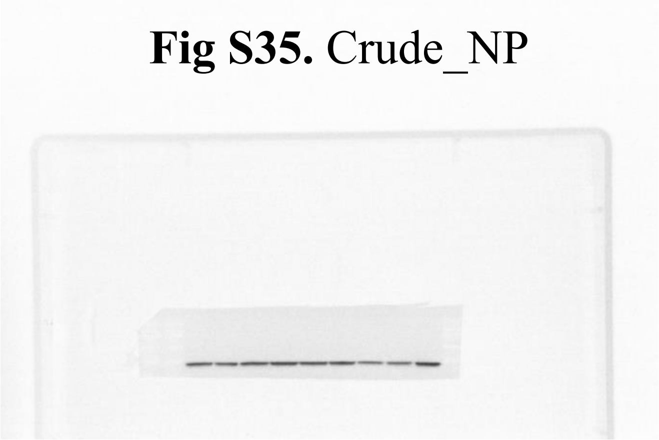

Fig. S35. Crude_beta-actin

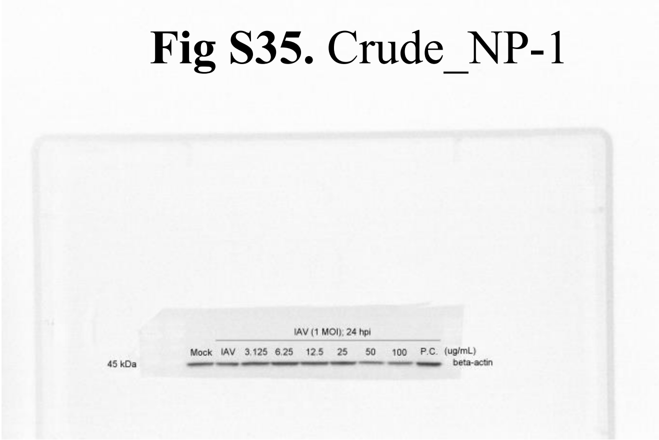

Fig. S35. Crude_beta-actin-1

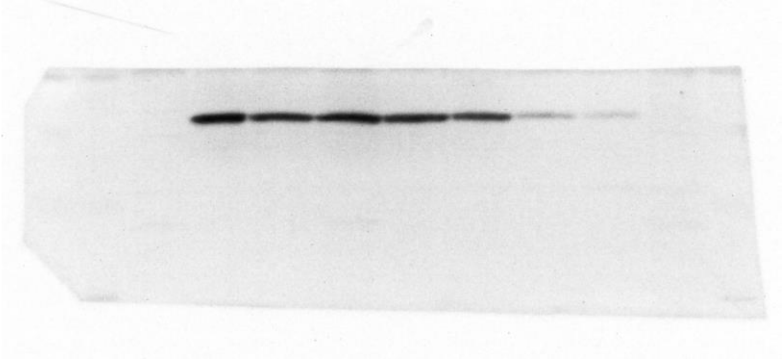

Fig. S35. Crude_NS1

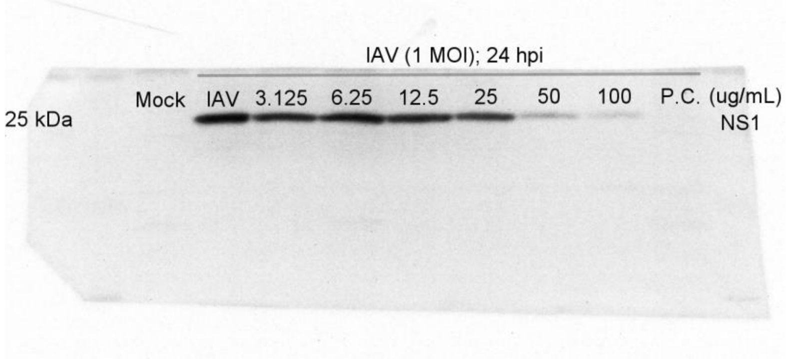

Fig. S35. Crude_NS1-1
